# China’s ambitious low-carbon goals require fostering city-level renewable energy transitions

**DOI:** 10.1016/j.isci.2023.106263

**Published:** 2023-02-24

**Authors:** Guanglei Yang, Guoxing Zhang, Dongqin Cao, Donglan Zha, Bin Su

**Affiliations:** 1 School of Management, Lanzhou University, Lanzhou 730000, China; 2 College of Economics and Management, Nanjing University of Aeronautics and Astronautics, Nanjing 211106, China; 3 Research Centre for Soft Energy Science, Nanjing University of Aeronautics and Astronautics, Nanjing 211106, China; 4 Energy Studies Institute, National University of Singapore, Singapore 119620, Singapore

**Keywords:** Energy resources, Energy policy, Energy systems, Energy management, Energy modeling

## Abstract

Cities in China, as elsewhere, are increasingly playing a crucial role in mitigating climate change. We developed a panel dataset on renewable energy transition in Chinese cities, and assessed the CO_2_ emissions reduction of city-level renewable energy transition. We found that city-level renewable energy transition only reduced 446 million tonnes of CO_2_ emissions from 2005 to 2019. Moreover, the 2030 carbon peak target will be missed in the business-*as*-usual scenario. The CO_2_ emissions reduction of city-level renewable energy transition will significantly increase in the policy constraint scenario and in the technology breakthrough scenario, and the 2030 carbon peak target will likely be reached in both these scenarios, with a range of possible CO_2_ emissions in 2030 equal to 8.34–10.43 and 8.00–10.07 billion tonnes, respectively. In this study, we were the first to assess the historical contribution and prospective trajectory of CO_2_ emissions reduction of China’s city-level renewable energy transition.

## Highlights

• We developed a panel dataset on the renewable energy transition in Chinese cities
• We assessed the CO_2_ reduction of city-level renewable energy transition in China
• Previous renewable energy transition in Chinese cities has not radically reduced CO_2_
• The city-level renewable energy transition is critical to carbon peaking in China

## Graphical abstract

**Figure:**
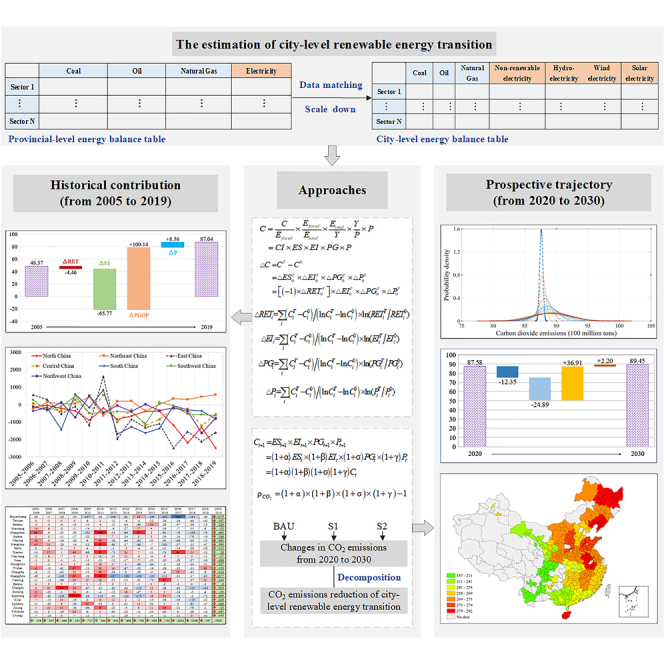

## Introduction

The transition to a predominant use of renewable energy is regarded as the most effective strategy to achieve the Paris Climate Agreement and the related Nationally Determined Contributions.^1^ As the largest contributor to global carbon emissions growth, China is taking a proactive role in addressing climate change. China has pledged to reach its carbon peak by 2030, with about 25% of its energy consumption coming from non-fossil fuels.^2^ In China, as elsewhere, renewable energy consumption (especially wind and solar) has experienced a rapid growth over the past decade, but it still accounted for less than 15% of total energy consumption in 2021.^3^ Even more worryingly, in 2020, China’s reserves-to-production ratios of coal, oil, and natural gas were equal to 37, 18, and 43 years, respectively.^3^ For these reasons, renewable energy transition will play an increasingly important role in China’s efforts to guarantee energy security.

The concept of renewable energy transition is derived from the sustainable development theory, the energy substitution theory, and the low-carbon economy theory. The sustainable development theory emphasizes not only the issues of sustainable economy, sustainable ecology, and sustainable society but also of sustainable energy,^4^ which focuses on the efficient use of fossil energy and the large-scale development of renewable energy. In the energy substitution theory, because of the scarcity of traditional fossil energy sources, their price will inevitably rise with their gradual depletion; this offers the possibility to replace fossil energy with renewable energy.^5^^,^^6^ In addition to the scarcity of fossil energy, external costs, caused by negative impacts such as environmental pollution and climate change, are constantly rising, providing an opportunity to replace fossil energy with renewable energy.^7^^,^^8^ According to the theory of low-carbon economy, renewable energy transition is expected to be a key driver of long-term carbon emissions reduction; in fact, its carbon emissions reduction effect is manifested mainly through the gradual replacement of traditional fossil energy with renewable energy.^9^^,^^10^ Theoretically, renewable energy transitions (i.e., the replacement of fossil energy by renewable energy) can reduce carbon emissions.

Renewable energy transition in China has been considered as an important tool to address climate change, as the ambitious low-carbon goals (i.e., carbon peaking by 2030 and carbon neutrality by 2060) call for renewable energy to dominate the energy system.^11^ In China, cities vary widely in terms of population, economic development, and carbon emissions. For example, in 2019, population ranged from 0.25 million people of Jiayuguan to 16.58 million people of Chengdu; the economic level ranged from a GDP per capita of ¥14,700 in Dingxi to ¥200,300 in Shenzhen^12^; and CO_2_ emissions ranged from 1.35 million tonnes of CO_2_ of Bazhong to 227.65 million tonnes of CO_2_ of Suzhou.^13^ Therefore, Chinese policymakers must gain a deeper understanding of the differences and characteristics of city-level renewable energy transition, as this is necessary to formulate efficient energy transition plans. As the largest global consumer of fossil energy, China has made great strides in carbon emission reduction, and cities are the basic administrative units to implement carbon emission reduction policies.^14^ No analysis has been performed to date of city-level renewable energy transition, which is far more complex than that at the national and provincial levels.

As efforts to reduce carbon emissions intensify, policymakers increasingly need more specific and detailed new measures to address climate change; city-level renewable energy transition can provide an excellent opportunity for this demand.^15^ However, studies on the impact of renewable energy transition on CO_2_ emissions only focus at the national and provincial levels, owing to data accessibility (see a list of representative studies in Table S1). For example, at the national level, Dong et al.^16^ analyzed the nonlinear impact of renewable energy transition on carbon emission performance in 32 developed countries, while Yuan et al.^10^ simulated CO_2_ emissions in 2025 under four different energy transition scenarios. At the provincial level, Xiao et al.^17^ simulated the CO_2_ emissions reduction effect under three different energy transition scenarios in the Beijing-Tianjin-Hebei region and the Yangtze River Delta region, while Lee et al.^18^ analyzed the impact of renewable energy development on carbon intensity in 30 Chinese provinces from 2000 to 2019. Compared to the national and provincial levels, city-level data on renewable energy transition are generally scarce and of low quality.

Although previous studies did not focus on the renewable energy consumption of Chinese cities, they covered other related city-level aspects, especially fossil energy consumption and CO_2_ emissions. For example, Mi et al.^19^ employed an input-output model to calculate the consumption-based CO_2_ emissions of 13 Chinese cities; and Shan et al.^15^ constructed a CO_2_ emission inventory for Chinese cities based on energy balance tables. The city-level data provided by the above-mentioned studies are all cross-sectional data. To reveal the dynamic characteristics of cities, the city-level data with time scales are further developed. For example, Cui et al.^20^ developed city-level industrial electricity consumption data from 1999 to 2014, and Shan et al.^14^ constructed a city-level CO_2_ emission inventory from 2001 to 2019. These pioneering studies have been widely emulated; a representative list is presented in Table S2. Undeniably, these studies lay a solid foundation for research on city-level renewable energy transition. However, these city-level relevant studies do not include data on renewable energy consumption, and city-level renewable energy transition cannot be measured by these methods. Moreover, China’s policymakers must monitor progress in renewable energy transitions at city level, and align the diversified regional efforts toward the ambitious low-carbon goals. Unfortunately, an assessment is currently missing of the historical contribution and prospective trajectory of CO_2_ emissions reduction through renewable energy transitions at the city level.

In view of the above-mentioned research gaps, in this study, we focused on three major aspects; our conceptual framework is summarized in Figure S1. Our study contributes to the existing literature in three ways: (a) based on the traditional provincial-level energy balance table (EBT), we decomposed electricity consumption into four components (i.e., non-renewable electricity, hydroelectricity, wind electricity, and solar electricity) to obtain an extended provincial-level EBT, which was scaled down to the city-level EBT, so as to assess city-level renewable energy transition; (b) we incorporated renewable energy transition into the Kaya identity to calculate its contribution to changes in city-level CO_2_ emissions from 2005 to 2019; and (c) we used the Monte Carlo technique to simulate the prospective trajectory of CO_2_ emissions reduction of city-level renewable energy transition in China from 2020 to 2030 under three different scenarios, namely the business-*as*-usual scenario (BAU), the policy constraint scenario (S1), and the technology breakthrough scenario (S2), thereby encompassing a wide range of possible future changes.

In this study, we investigated for the first time the historical contribution (from 2005 to 2019) and the prospective trajectory (from 2020 to 2030) of CO_2_ emissions reduction of China’s city-level renewable energy transition. The results showed that China’s city-level renewable energy transition progressed smoothly from 2005 to 2019, although only 446 million tonnes of CO_2_ emissions were reduced. From 2020 to 2030, without additional policy constraints or technological innovations, the CO_2_ emissions reduction from city-level renewable energy transition is expected to remain very low (only 510 million tonnes), and the 2030 carbon peak goal will be far away. In contrast, in both the S1 and S2 scenarios, the CO_2_ emissions reduction effect of city-level renewable energy transition will be significantly enhanced (equal to 907 and 1,235 million tonnes, respectively), and the 2030 carbon peak target will be likely reached. It is worth noting that the increase in CO_2_ reduction from city-level renewable energy transition will be larger in the S2 scenario (i.e., 328 million tonnes) compared to the S1 scenario. This means that city-level renewable energy transition will likely play an unparalleled role in China’s 2030 carbon peak target. Hence, we urge enhanced policy and innovation intensity, further promoting city-level renewable energy transition in the future.

## Results

### The peer-group characteristics of city-level renewable energy transition

**Figure 1:**
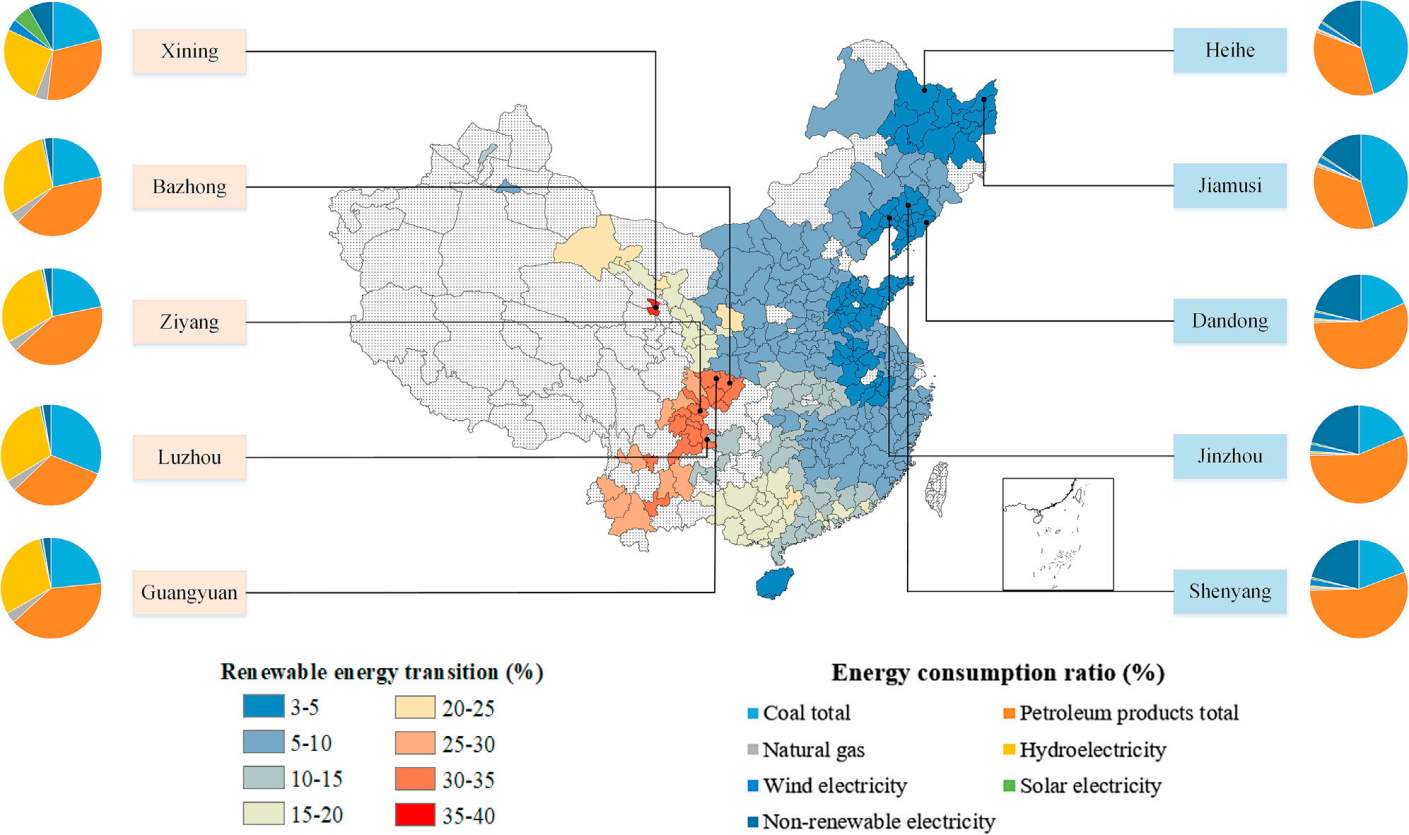
Spatial distribution of renewable energy transition of 279 Chinese cities in 2019, as well as the composition of their energy consumption of the top five cities in terms of renewable energy transition (left pie chart) and the bottom five cities in terms of renewable energy transition (right pie chart)

In this study, the proportion of hydroelectricity, wind electricity, and solar electricity consumption in total energy consumption was used to characterize the city-level renewable energy transition. The spatial distribution of renewable energy transition in 279 Chinese cities in 2019 is presented in Figure 1. In 2019, the average share of renewable energy consumption (i.e., hydroelectricity, wind electricity, and solar electricity) in 279 Chinese cities was only 10.90%. More in detail, renewable energy consumption was lower than 15% in 220 of the 279 cities investigated and higher than 30% only in 18 cities. This means that there is a considerable room to perform city-level renewable energy transition in China.

Heihe, Jiamusi, Dandong, Jinzhou, and Shenyang were the five cities with the lowest share of renewable energy consumption, with more than 95% of energy consumption coming from coal, oil, natural gas, and non-renewable electricity. Xining, Bazhong, Ziyang, Luzhou, and Guangyuan were the five cities with the highest share of renewable energy consumption. Different from cities with a low proportion of renewable energy consumption, in these cities, hydroelectricity consumption had a relatively high proportion in total energy consumption, accounting for 26.15%, 30.36%, 30.03%, 29.99%, and 29.97%, respectively.

Moreover, China’s city-level renewable energy transition showed to be higher in the Western region and lower the in Eastern region. In fact, those cities with a high proportion of renewable energy consumption were mainly concentrated in the southwestern and northwestern regions of China, especially Sichuan, Yunnan, Qinghai, Gansu, and Guangxi. In contrast, cities with a lower proportion were mainly located in Northeast China, North China, and Eastern China, especially in Heilongjiang, Liaoning, Shandong, and Anhui.

### The historical contribution of CO_2_ emissions reduction of city-level renewable energy transition

**Figure 2:**
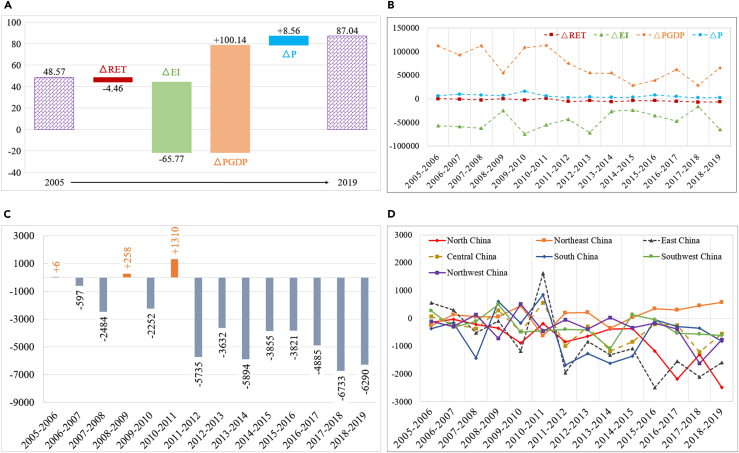
Decomposition of driving factors of CO_2_ emissions in China from 2005 to 2019 (A) Cumulative decomposition results of changes in each driving factor (unit: 100 million tonnes); (B) historical trend of decomposition results for each driving factor (unit: 10 thousand tonnes); (C) historical trend of decomposition results of changes in city-level renewable energy transition (unit: 10 thousand tonnes); and (D) historical trend of decomposition results of city-level renewable energy transition changes in different regions (unit: 10 thousand tonnes).

Figure 2 presents the decomposition results of the driving factors of the change of CO_2_ emissions in China from 2005 to 2019. As shown in Figure 2A, in 2019, CO_2_ emissions increased by 3.847 billion tonnes compared to 2005. During this period, energy intensity and economic scale were the largest driving factors for carbon emission reduction and increase, respectively, the former reducing CO_2_ emissions by 6.577 billion tonnes and the latter increasing them by 10.014 billion tonnes. These results are consistent with the findings of Wang et al.^21^ and Zha et al.^22^ From 2005 to 2019, China’s relatively slow population growth resulted in 856 million tonnes of CO_2_ emissions. City-level renewable energy transition contributed to the relatively small amount of 446 million tonnes of CO_2_ emissions reduction. In addition, the CO_2_ emissions reduction driven by city-level renewable energy transition had only a small growth rate over time (see Figure 2B). This indicates that city-level renewable energy transition did not play a dominant role in CO_2_ emissions reduction during the period from 2005 to 2019. This may be due to the fact that, first, in 2019, the share of renewable energy consumption was relatively small 10.88%; second, renewable energy consumption increased relatively slowly, with its share in total energy consumption increasing only from 4.42% in 2005 to 10.88% in 2019.

The city-level renewable energy transition did not reduce CO_2_ emissions in all years. For example, it increased CO_2_ emissions by 0.06 million tonnes in 2008–2009, by 2.58 million tonnes in 2008–2009, and by 13.10 million tonnes in 2010–2011. This was mainly due to the rebound in total fossil energy consumption and the negative growth in the city-level renewable energy transition in these three periods (see Figure 2C). Specifically, in 2005–2006, the share of renewable energy consumption (i.e., hydroelectricity, wind electricity, and solar electricity) in total energy consumption (i.e., coal, oil, natural gas, nuclear electricity, hydroelectricity, wind electricity, and solar electricity) in China decreased from 5.441% to 5.435%, resulting in an increase of 0.06 million tonnes of CO_2_ emissions.^3^ Similarly, in 2008–2009 and 2010–2011, the share of renewable energy consumption decreased by 0.401% and 0.543%, respectively, which is the possible reason for the increase of CO_2_ emissions observed in these two periods.^3^

Significant differences were found in the CO_2_ emissions reduction from city-level renewable energy transition across regions. As shown in Figure 2D, this effect was relatively large in North China and East China, with −1.39 and 5.60 million tonnes in 2005–2006 and −24.84 and −15.97 million tonnes in 2018–2019, respectively. In contrast, the CO_2_ emissions reduction from city-level renewable energy transition decreased in Northeast China, while it remained relatively stable in the Southwest China.

**Figure 3:**
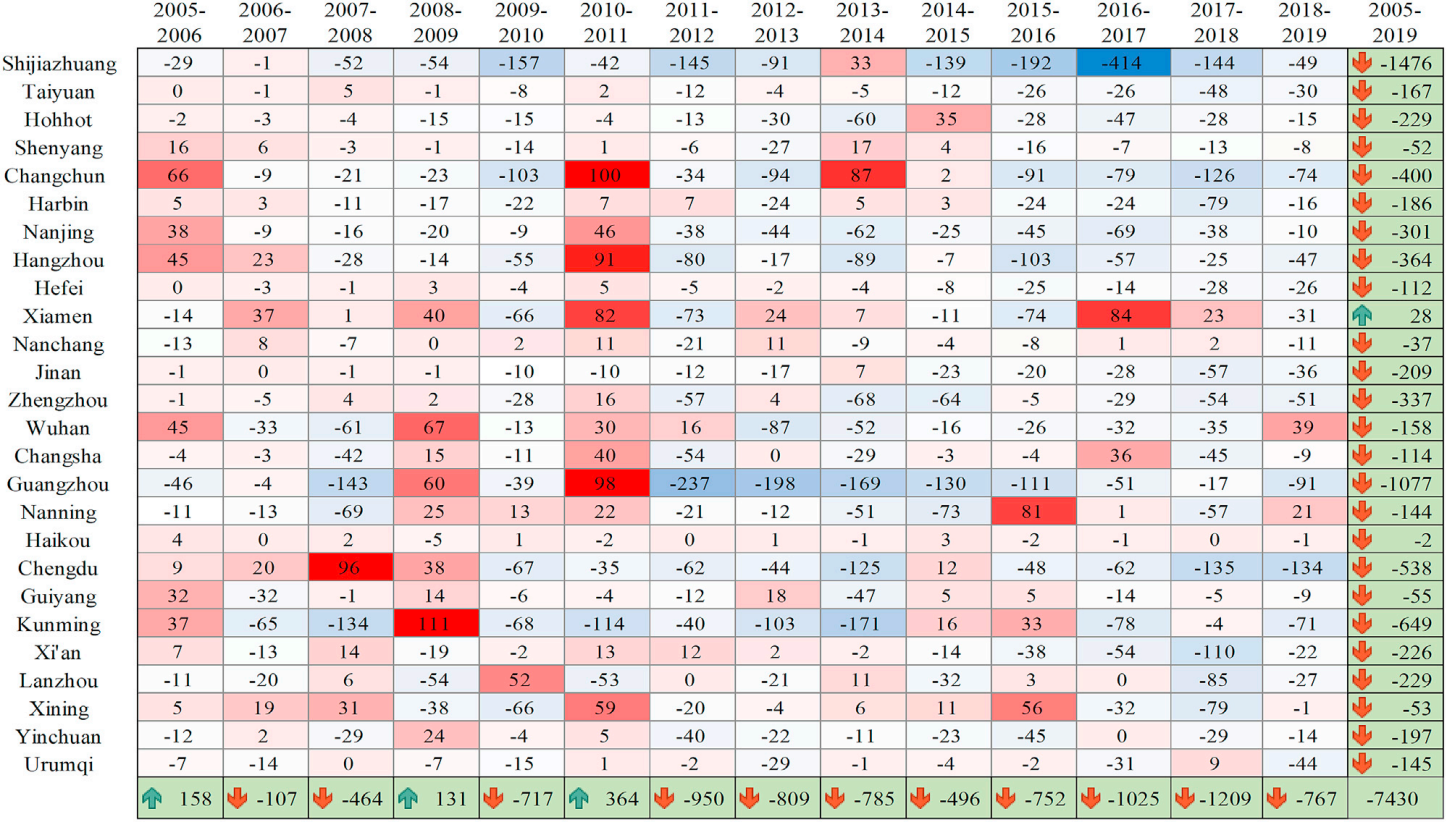
CO_2_ emissions reduction of renewable energy transition in representative cities from 2005 to 2019 (unit: 10,000 tonnes)

Figure 3 shows the CO_2_ emissions reduction from renewable energy transition in representative cities. In this study, we selected 2005–2006, 2012–2013, and 2018–2019 as representative periods for analysis. As shown in Figure 3, the renewable energy transition in 2005–2006 entailed an increase in CO_2_ emissions in 12 cities. For example, the renewable energy transition in Changchun, Hangzhou, and Wuhan resulted in an increase in CO_2_ emissions of more than 0.4 million tonnes. In contrast, the renewable energy transition in most cities reduced CO_2_ emissions to varying degrees. During this period, Guangzhou was the city with the largest CO_2_ emissions reduction from renewable energy transition, equal to 0.46 million tonnes. From 2012 to 2013, the renewable energy transition in Xiamen, Guiyang, Hefei, Zhengzhou, and Xi’an generated an increase in CO_2_ emissions. This indicates that, compared to 2005–2006, the proportion of renewable energy consumption in most cities in 2012–2013 increased to varying degrees. More in detail, renewable energy transition in Guangzhou resulted in the largest CO_2_ emissions reduction, equal to 2.37 million tonnes. During the period from 2018 to 2019, the renewable energy transition of Wuhan and Nanjing resulted in an increase in CO_2_ emissions, while in all other cities it reduced CO_2_ emissions to varying degrees. Specifically, Chengdu surpassed Guangzhou to become the city with the highest amount of CO_2_ emissions reduction through renewable energy transition, equal to 1.34 million tonnes of CO_2_ emissions.

Overall, 20 of these cities reduced in total more than 1 million tonnes of CO_2_ emissions from renewable energy transition, and 12 of them achieved more than 2 million tonnes of CO_2_ reductions. Among these, the highest CO_2_ emissions reduction from renewable energy transition was recorded in Shijiazhuang, with a reduction of 14.76 million tonnes, followed by Guangzhou and Kunming, with a reduction of 10.77 and 6.49 million tonnes, respectively. Interestingly, among all representative cities, only Xiamen’s renewable energy transition resulted in an increase in CO_2_ emissions from 2005 to 2019 (as did all cities in Fujian). The possible reason is that in Fujian, the share of renewable electricity consumption in total energy consumption decreased from 16.7% in 2005 to 11.4% in 2019; more specifically, in 2010–2011 and 2016–2017, it decreased from 15.6% to 9.4% and from 17.7% to 12.1%, respectively.^23^

### The prospective trajectory of CO_2_ emissions reduction of city-level renewable energy transition

Based on the factor growth rates set in the three scenarios considered (see Table S6), we first used the Monte Carlo simulation technique to perform 500,000 random self-sampling. Then, the average annual growth rate of CO_2_ emissions was calculated, and the evolution trend of CO_2_ emissions under different scenarios from 2020 to 2030 was simulated. To assess the robustness of the simulation results, we extended the time horizon of the scenario simulation to 2035, and compared the results from 2020 to 2030 with those from 2031 to 2035.

**Figure 4:**
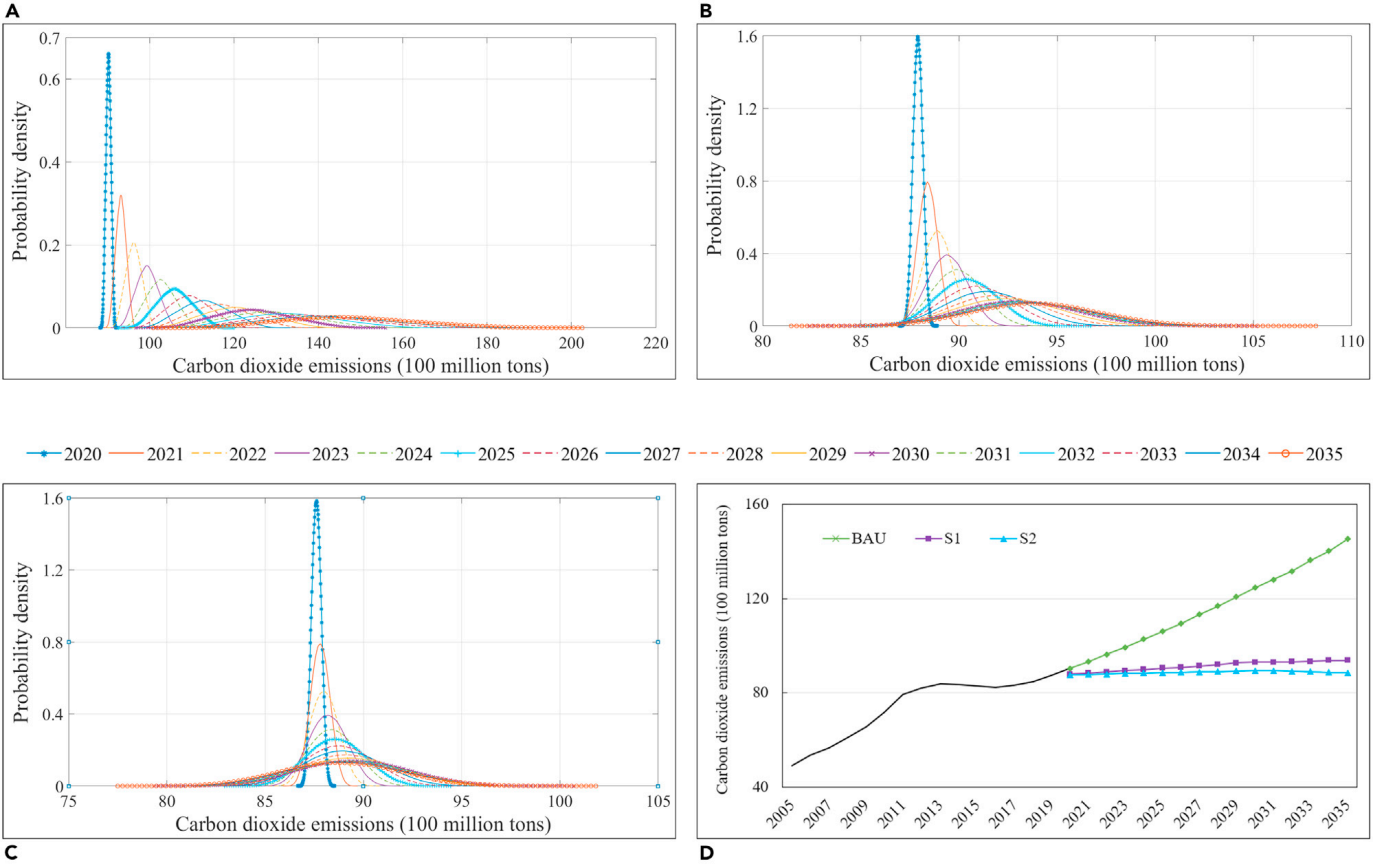
Evolution trend of city-level CO_2_ emissions in China from 2020 to 2035 (A) BAU scenario, (B) S1 scenario, (C) S2 scenario, and (D) historical trends and prospective trajectory.

In the BAU scenario, i.e., without additional policy constraints or significant technological innovations, China’s CO_2_ emissions will continue to increase substantially from 2020 to 2030 (see Figure 4A) ). Specifically, the range of CO_2_ emissions in 2020 was projected from 8.83 to 9.21 billion tonnes, with the highest probability of 9.03 billion tonnes. By 2025, CO_2_ emissions will range from 9.26 to 11.96 billion tonnes, with an average annual growth rate of 0.97%–5.97% compared to 2020. CO_2_ emissions in 2030 are likely to increase further, ranging from 9.72 to 15.51 billion tonnes, with the highest probability of 12.46 billion tonnes. Compared to 2020, the average annual growth rate will be from 1.01% to 6.84%. Moreover, the range of possible CO_2_ emissions in 2030 will not fully encompass the range of possible emissions before 2030. In the BAU scenario, CO_2_ emissions will increase from 12.46 billion tonnes in 2030 to 14.52 billion tonnes in 2035. This means that, if current plans to reduce CO_2_ emissions are not optimized enough, the CO_2_ emissions will be higher and there will be a greater probability of missing the 2030 carbon peak target.

In the S1 scenario, the growth rate of CO_2_ emissions will slow down significantly (see Figure 4B). Specifically, the range of CO_2_ emissions in 2025 and 2030 will be 8.52–9.63 billion tonnes and 8.34–10.43 billion tonnes, respectively. Compared to 2020, the ranges of the average annual growth rate will be −0.41% to 1.69% and −0.41% to 1.75%, respectively. Obviously, under the policy constraints, the growth rate of CO_2_ emissions will be effectively suppressed. From the evolution trend of CO_2_ emissions, the range of CO_2_ emissions in 2030 (from 8.34 to 10.43 billion tonnes) will include the range of CO_2_ emissions in other years (e.g., from 8.28 to 10.34 billion tonnes in 2029 and 8.33 to 10.17 billion tonnes in 2028), which indicates that CO_2_ emissions are likely to peak in 2030 under the S1 scenario. However, from the scope of the envelope, the 2030 CO_2_ emissions peak target will still be very uncertain. For each year from 2031 to 2035, CO_2_ emissions will be 9.305, 9.314, 9.334, 9.378, and 9.382 billion tonnes, respectively. The probability that CO_2_ emissions for each year in 2031–2035 will be lower than those in 2030 is 47.8%, 45.5%, 44.0%, 41.3%, and 39.5%, respectively (see Table S7). Although the 2030 carbon peak target is likely to be achieved under the S1 scenario, there is still a greater than 50% probability of a slow increase in CO_2_ emissions from 2031 to 2035. This means that additional drivers will be needed to ensure that the 2030 carbon peak target is reached.

In the S2 scenario, CO_2_ emissions will be further reduced compared to the S1 scenario (see Figure 4C). Specifically, the range of CO_2_ emissions in 2025 and 2030 will be 8.33–9.45 billion tonnes and 8.00–10.07 billion tonnes, respectively, and the corresponding average annual growth rates will be −0.78% to 1.33% and −0.77% to 1.37%, respectively. This indicates that the scale of China’s CO_2_ emissions will shrink further under policy constraints and significant technological breakthroughs (see Figure 4D). The range of CO_2_ emissions in 2030 (from 8.00 to 10.07 billion tonnes) will completely envelop the range of CO_2_ emissions in other years (e.g., from 8.01 to 9.94 billion tonnes in 2029 and 8.08 to 9.81 billion tonnes in 2028), and the most likely CO_2_ emissions in 2029 (8.93 billion tonnes) will be very close to the most likely CO_2_ emissions in 2030 (8.95 billion tonnes). Moreover, CO_2_ emissions will decrease slightly from 2031 to 2035, and will be equal to 8.94, 8.92, 8.89, 8.87, and 8.86 billion tonnes, respectively. The probability that CO_2_ emissions for each year in 2031–2035 will be lower than those in 2030 is 50.2%, 52.0%, 53.8%, 55.5%, and 57.0%, respectively (see Table S7). Compared to the S1 scenario, under the S2 scenario, there is a greater than 50% probability of a slow decrease in CO_2_ emissions from 2031 to 2035. This means that in the S2 scenario, total CO_2_ emissions will have a high probability of peaking before 2030.

Among these three scenarios, the target of carbon peak will likely be achieved in 2030 in both S1 and S2 scenarios. This is consistent with the findings of previous studies. For example, Qi et al.^24^ found that under the policy scenario, the carbon peak target would be achieved during the period 2024–2033, with CO_2_ emissions ranging from 8.74 to 9.62 billion tonnes. Moreover, Zhang et al.^25^ and Liu et al.^26^ found that China was on track to meet its carbon peak target by 2030 under the scenario of energy structure adjustment, and total CO_2_ emissions in 2030 were calculated as between 10.0 and 11.0 billion tonnes and between 9.5 and 10.5 billion tonnes, respectively. In the BAU scenario, the promotion of renewable energy transition, the improvement of energy efficiency, the increase of the GDP per capita, and the expansion of population will not be enough to achieve the carbon peak target in 2030. Similarly, Wu and Xu^27^ found that rapid economic growth offsets the carbon emission reduction effects of technological progress and energy structure optimization, and CO_2_ emissions would reach about 12.5 billion tonnes in 2030. Therefore, it is necessary to strengthen policy implementation and increase technological innovation investment to achieve the 2030 carbon peak target.

**Figure 5:**
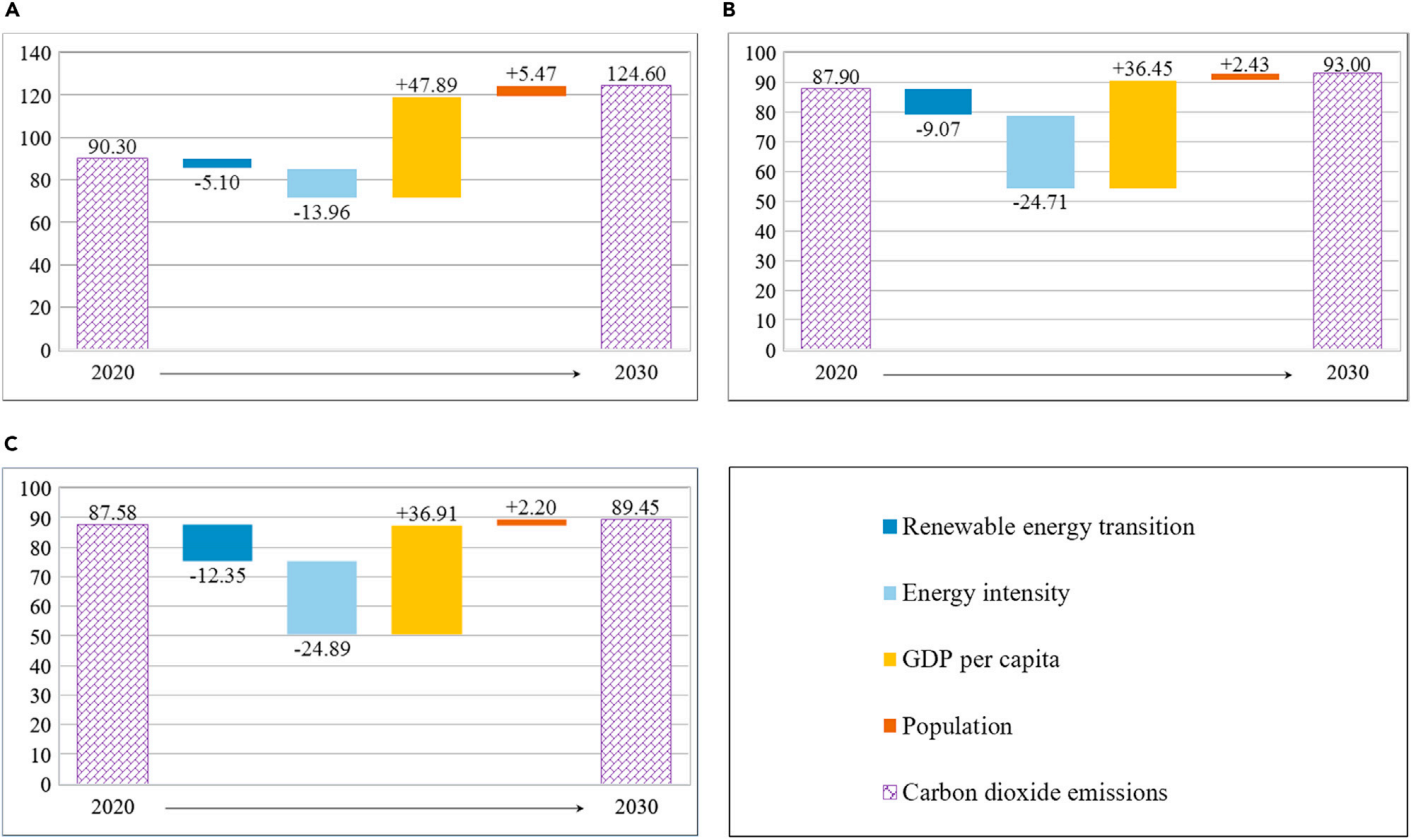
Driver decomposition of CO_2_ emissions in China from 2020 to 2030 (unit: 100 million tonnes) (A) BAU scenario, (B) S1 scenario and (C) S2 scenario.

The scenario simulation results show that the carbon peak target before 2030 will likely be reached in both S1 and S2 scenarios, and that the latter has a stronger CO_2_ emissions reduction effect than the former. To grasp the contribution of the driving factors of CO_2_ emissions reduction under the three different scenarios, the driving factors of the overall CO_2_ emissions reduction were further decomposed; the differences are shown in Figure 5. In the BAU scenario, the total CO_2_ emissions will increase by 3.430 billion tonnes from 2020 to 2030, with economic scale and population size entailing an increase by 4.789 and 0.547 billion tonnes, respectively, and renewable energy transition and energy intensity entailing a decrease by 0.510 and 1.396 billion tonnes, respectively. In contrast, the CO_2_ emissions reduction effect of renewable energy transition will be relatively weak (see Figure 5A). Compared to the BAU scenario, in the S1 scenario, the CO_2_ emissions reduction effects of both economic scale and population size will be weakened, entailing a reduction of 3.645 and 0.243 billion tonnes, respectively. Conversely, both the renewable energy transition and energy intensity will generate significant increases in CO_2_ emissions reduction, by 0.907 and 2.471 billion tonnes, respectively (see Figure 5B).

In the S2 scenario, the total CO_2_ emissions will increase from 8.758 billion tonnes in 2020 to 8.945 billion tonnes in 2030. More in detail, economic scale and population size will increase CO_2_ emissions by 3.691 and 0.220 billion tonnes, respectively, while energy intensity and renewable energy transition will reduce CO_2_ emissions by 2.489 and 1.235 billion tonnes, respectively. Interestingly, in the S2 scenario, the CO_2_ emissions reduction of economic scale, population size, and energy intensity will basically have no significant change compared to the S1 scenario, entailing only an increase of 46 million tonnes, a decrease of 23 million tonnes, and a decrease of 18 million tonnes, respectively. However, in the S2 scenario, renewable energy transition will generate an increase of CO_2_ emissions reduction by −328 million tonnes compared to the S1 scenario (see Figure 5C). Moreover, the CO_2_ emissions reduction from renewable energy transition, energy intensity, economic scale, and population size under the S2 scenario will be 2.43 times, 1.79 times, 0.77 times, and 0.40 times those under the BAU scenario, respectively. This also means that renewable energy transition is expected to play a critical role for reaching the 2030 carbon peak target, with considerable scope for emission reductions.

**Figure 6:**
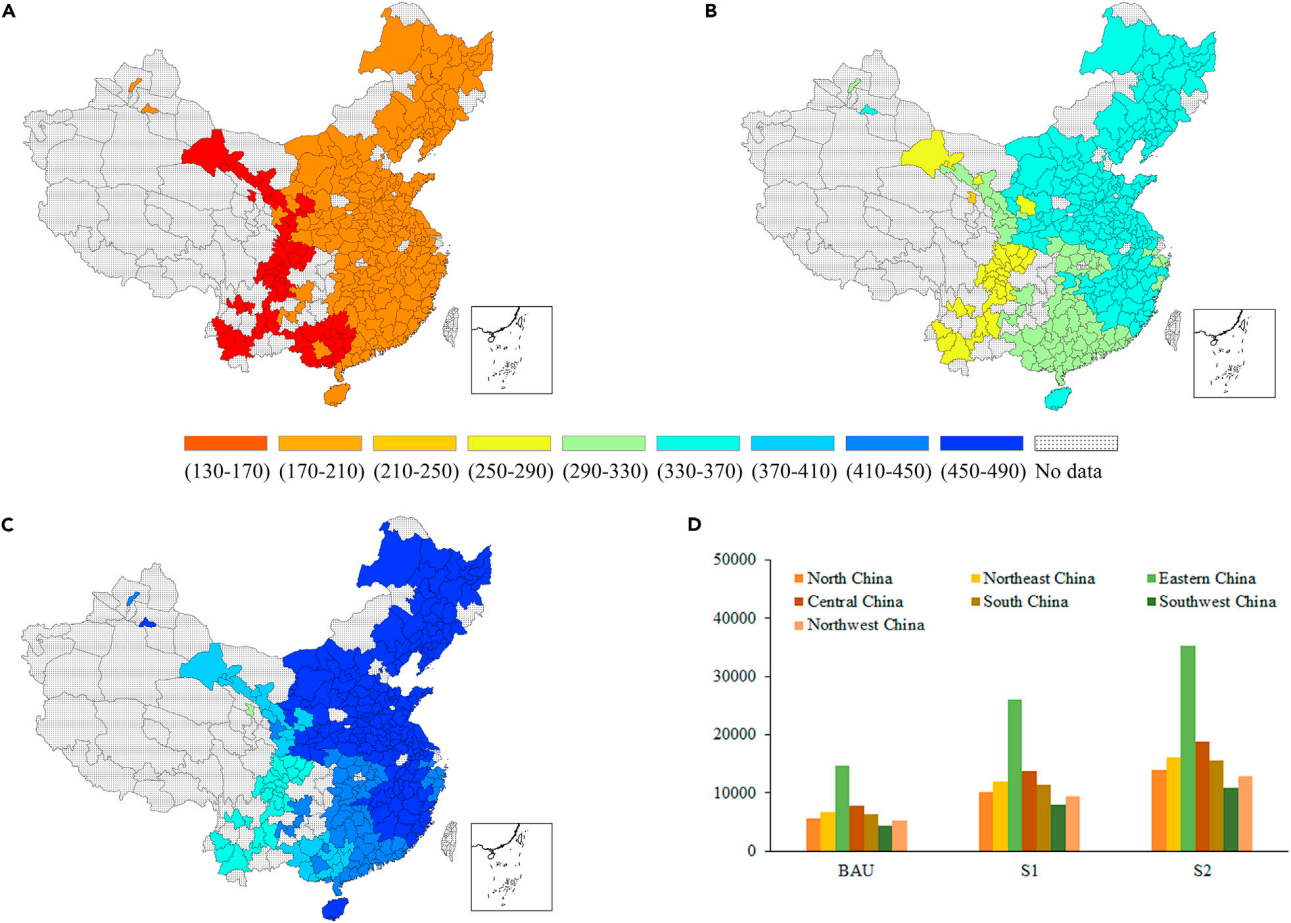
CO_2_ emissions reduction (CERs) of city-level renewable energy transition from 2020 to 2030 (unit: 10 thousand tonnes) (A) BAU scenario, (B) S1 scenario, (C) S2 scenario, (D) regional emission reductions under different scenarios.

We then decomposed the CO_2_ emissions reduction from renewable energy transition at the city level (see Figure 6). Obviously, the CO_2_ emissions reduction effect of city-level renewable energy transition in the S2 scenario will be significantly stronger than that in the S1 scenario, which in turn will be obviously stronger than that in the BAU scenario. Specifically, in the BAU scenario, no city will have a CO_2_ emissions reduction of more than 2.50 million tonnes, and the difference between cities will be small, with 236 of the 279 cities having a CO_2_ emissions reduction between 2.00 and 2.36 million tonnes (see Figure 6A). This means that under the BAU scenario, the CO_2_ emissions reduction effect of city-level renewable energy transition will be relatively weak. Compared to the BAU scenario, in the S1 scenario, the CO_2_ emissions reduction effect of city-level renewable energy transition will significantly increase. The CO_2_ emissions reduction of all cities will be between 2.82 and 4.26 million tonnes, and 167 cities will have a reduction of more than 4.00 million tonnes (see Figure 6B). The CO_2_ emissions reduction effect of city-level renewable energy transition in the S2 scenario will further improve compared to the S1 scenario, ranging from 3.82 to 5.75 million tonnes, and 225 cities will have a CO_2_ emissions reduction of more than 5.00 million tonnes (see Figure 6C).

Significant peer-group characteristics were revealed in the analysis of the CO_2_ emissions reduction effect of city-level renewable energy transition. In all scenarios, the CO_2_ emissions reduction effect of renewable energy transition in cities in the Southwest China, such as Chengdu and Kunming, will be significantly weaker than that in other regions. However, the CO_2_ emissions reductions of city-level renewable energy transition in South China and Central China under the S1 and S2 scenarios will be significantly lower than that in North China, East China, and Northeast China. Among the seven regions investigated, the CO_2_ emissions reduction of city-level renewable energy transition will be highest in Eastern China, and lowest in Southwest China (see Figure 6D). For example, in the S2 scenario, the CO_2_ emissions reduction in East China will be equal to 423.44 million tonnes, followed by Central China with 225.82 million tonnes; Southwest China will have the lowest level, equal to only 129.49 million tonnes.

### The CO_2_ emissions reduction potential of city-level renewable energy transition

**Figure 7:**
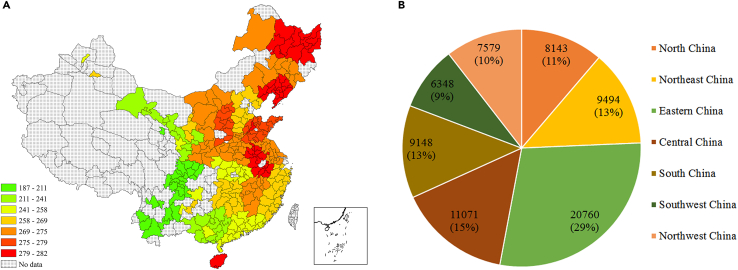
CERs potentials of city-level renewable energy transition from 2020 to 2030 (unit: 10 thousand tonnes) under the S2 scenario (A) in 279 Chinese cities and (B) in seven regions of China.

Furthermore, we assessed the CO_2_ emissions reduction (CERs) potential of city-level renewable energy transition. According to the simulation results, the CO_2_ emissions reduction effect of city-level renewable energy transition still has considerable room for improvement. The CO_2_ emissions reduction effect of city-level renewable energy transition under the S1 and S2 scenarios will be significantly stronger than that under the BAU scenario. Therefore, to a large extent, the CERs potentials of city-level renewable energy transition will be affected by the intensity of environmental policies and the level of technological innovation. Following Zhang et al.,^28^ the CO_2_ emissions reduction potential of the renewable energy transition in the S2 scenario can be defined as the CO_2_ emissions reductions from the S2 scenario relative to the BAU scenario, as shown in Figure 7.

We can see that the renewable energy transition of all cities has a CO_2_ emissions reduction potential of at least 2.25 million tonnes, and that of 71 cities, such as Taiyuan, Shenyang, and Harbin, exceeds 3.30 million tonnes (see Figure 7A). The total CO_2_ emissions reduction potential of renewable energy transition under the S2 scenario is about 725 million tonnes. Therefore, we can confirm that under the S2 scenario, city-level renewable energy transition has considerable potential for carbon emissions reduction.

Moreover, the CERs potential of renewable energy transition varies significantly across cities (see Figure 7B). For example, Eastern China has the largest CO_2_ emissions reduction potential, with 249.46 million tonnes of CO_2_ reduction, accounting for 29% of total emission reduction, followed by Central China, accounting for 15% of the total emission reduction. In contrast, the renewable energy transition in Southwest China has the smallest emission reduction potential, equal to 76.28 million tonnes of CO_2_ emissions and accounting for only 9% of the total emission reduction.

## Discussion

### Uncertainty analysis

The purpose of the uncertainty analysis was to identify the limitations and potential uncertainties of the estimated city-level renewable energy transition, so as to provide a forward-looking statement for subsequent more accurate calculations of city-level renewable energy transition. In this study, significant uncertainties were found to exist in China’s city-level renewable energy transition estimates, in particular in the compilation of the city-level EBT and in the activity data related to socio-economic factors.

First, there were uncertainties in China’s city-level energy consumption accounting, mainly caused by the lack of EBTs in cities. No city in China has access to EBTs or final energy consumption data for the period 2005–2019. In this context, based on the approach of Shan et al.,^29^ we used socioeconomic indexes (e.g., GDP, industrial output, urban population, and rural population) to downscale provincial data to the city level, and then developed the city-level EBTs. The assumption of this approach was that each city has the same per capita energy consumption and economic structure as the respective province. However, in fact, the per capita energy consumption and economic structure of different cities in the same province are different from some extent.

Second, there were uncertainties in the socio-economic indexes, mainly caused by differences in the statistical caliber and the incompleteness of data. In the provincial-level EBT, the final energy consumption of seven industries (i.e., *Agriculture*, *Forestry*, *Animal Husbandry and Fishery*, *Industry*, *Construction*, *Transport*, *Storage and Post*, *Wholesale and Retail Trades*, *Hotels and Catering Services*, *Other services*, and *Residential*) was comprehensively counted. Unfortunately, for more than 80% of the cities, the GDP or population data of these seven industries cannot be obtained, and only partial data can be obtained (for example, for cities in Fujian, only the GDP of three industries can be retrieved). Therefore, we were forced to downscale the city-level data roughly from the provincial level.

### Verification

To our knowledge, there is no effective method to quantify these uncertainties. What is certain, however, is that the approach of this study is reliable for the purpose to obtain city-level EBT, based on several precedents in city-level CO_2_ emissions accounting.^30^^,^^14^ To verify the accuracy of city-level renewable energy transition, we conducted a verification with existing relevant data. In fact, no studies have been found that provide city-level data on renewable energy transitions. Theoretically, fossil fuels are the largest source of CO_2_ emissions, with coal, oil, and gas consumption accounting for more than 93% of total CO_2_ emissions in China in 2020.^31^ This indicates that fossil energy consumption and CO_2_ emissions are highly coupled.

Therefore, we used the city-level fossil energy consumption estimated in this study from 2005 to 2017 and the city-level CO_2_ emissions published in the CEADs database to conduct the intraclass correlation coefficients (ICC) test; the results are reported in Table 1. As shown in Table 1, the ICC of the individual was 0.895, which is significantly different from 0 at 95% confidence interval, while that of the average was as high as 0.944 and passed the 1% significance level test. Both test results indicate that the city-level fossil energy consumption estimated in this study had a strong, consistent relationship with city-level CO_2_ emissions. This indicates that city-level renewable energy transition in this study was relatively accurate and our approach was valid and reliable.

**Table 1:** ICC test of city-level fossil energy consumption and CO_2_ emissions

One-way random-effects model	ICC	p value	95%CI (L)	95%CI (U)
Individual ICC(C, 1)	0.895^∗∗^	0.025	0.501	0.984
Average ICC(C, K)	0.944^∗∗∗^	0.009	0.667	0.992

Note: ∗∗ and ∗∗∗ indicate 5% and 1% significance levels, respectively.

### Conclusions

In this study, we used a computational modeling technique to calculate the renewable energy transition of 279 Chinese cities from 2005 to 2019. Based on this, we traced the historical contribution of CO_2_ emissions reduction of city-level renewable energy transition through the Kaya identity. Moreover, we set three scenarios to cover the most likely direction of future CO_2_ emissions, and used the Monte Carlo technique to dynamically simulate China’s CO_2_ emissions from 2020 to 2030. Finally, the prospective trajectory of CO_2_ emissions reduction of city-level renewable energy transition was determined.

Our study showed that, first, there was a great heterogeneity in the renewable energy transition of 279 Chinese cities. The majority of cities in Southwest China and Northwest China are leaders in renewable energy transition, while those in Northeast China, North China, and Eastern China are still at a low level of renewable energy transition. Therefore, we urge that these differences should be fully incorporated into the formulation and implementation of China’s renewable energy transition policies. Specifically, although North China and Eastern China are economically developed regions, their hydroelectricity resources endowment is low, especially in cities such as Nanjing, Qingdao, and Suzhou. This means that wind electricity and solar electricity are likely to play an increasingly important role in the energy transition in the future, and specific strategies to ensure their development are urgently needed. In addition, although Southwest China is rich in hydroelectricity resources, cities in Eastern China are leading in hydroelectricity technology. Therefore, we propose deeper cooperation in hydroelectricity technology between cities in Southwest China and in Eastern China to further improve the efficiency of hydropower generation and utilization, especially in representative cities such as Chengdu and Kunming.

Second, although renewable energy transition only reduced 446 million tonnes of CO_2_ emissions from 2005 to 2019, the differences in contribution between cities should be seriously considered. For example, Guangzhou’s renewable energy transition reduced CO_2_ emissions by 10.77 million tonnes from 2005 to 2019, while Xiamen’s renewable energy transition increased carbon dioxide emissions by 0.28 million tonnes. Therefore, we propose that the actual CO_2_ emissions reduction from the renewable energy transition in different cities should be objectively assessed. Moreover, there are broad differences in the CO_2_ emissions reduction of city-level renewable energy transition across different periods. For example, the renewable energy transition in Shijiazhuang reduced CO_2_ emissions by 4.14 million tonnes in the period 2016–2017, but increased CO_2_ emissions by 0.33 million tonnes in the period 2013–2014. This finding warns that the dynamic differences in the CO_2_ emissions reduction of city-level renewable energy transition should be fully considered in policy formulation.

Third, the scenario simulation results showed that in the S1 scenario the CO_2_ emissions reduction from city-level renewable energy transition will significantly increase compared to the BAU scenario, and the same is true in the S2 scenario compared to the S1 scenario. Differently, compared to the S1 scenario, the increase in CO_2_ emissions reduction from renewable energy transition in the S2 scenario will be considerably higher than that from other drivers. This means that city-level renewable energy transition may play a pivotal role in reaching the carbon peak by 2030. Therefore, we propose to implement effective policy constraints and increase the intensity of investment in renewable energy technological innovation during 2020–2030 to fully leverage the CO_2_ emissions reduction effect of city-level renewable energy transition. In both the S1 and S2 scenarios, the CERs potential of renewable energy transition in cities in Northeast China, such as Harbin and Shenyang, and in cities in Eastern China, such as Qingdao and Hefei, is significantly higher than that of cities in Northwest China and Southwest China, such as Chengdu, Kunming, and Xining. This indicates that policy constraints and renewable energy technological innovation should be favored in cities in Northeast and Eastern China.

### Limitations of the study

In this study, we tried our best to ensure that the results are accurate and credible; however, there are still some limitations and uncertainties in relation to the following two aspects. **First**, the calculation of city-level renewable energy transition ignores the heterogeneity across cities. For example, in the deduction process of city-level EBTs, we used socio-economic indexes as the link between provinces and cities; however, energy structure and industrial structure are often different across cities. Therefore, in future research, we should optimize computational modeling techniques to provide a more detailed city-level energy balance table covering more than 30 energy categories and more than 40 socioeconomic sectors. **Second**, scenarios S1 and S2 are the most likely future directions for CO_2_ emissions reduction. However, considering the impacts of COVID-19 and other external factors, there may be more complex development scenarios for CO_2_ emissions reduction in 2020–2030. Therefore, we should fully consider the other potential development directions of CO_2_ emissions in future work, with a view to provide a more comprehensive and detailed prospective trajectory of CO_2_ emissions reduction.

## STAR★Methods

### Key resources table

**Table:** 

REAGENT or RESOURCE	SOURCE	IDENTIFIER
**Deposited data**		

National-level renewable energy consumption	Statistical Review of World Energy	https://www.bp.com/content/dam/bp/business-sites/en/global/corporate/xlsx/energy-economics/statistical-review/bp-stats-review-2022-all-data.xlsx
Provincial-level Energy Balance Table	China Energy Statistical Yearbook	http://www.stats.gov.cn/tjsj/tjcbw/
Provincial-level renewable energy production	China Electric Power Yearbook	http://www.stats.gov.cn/tjsj/tjcbw/
Provincial-level renewable energy consumption in 2019	Renewable Energy Power Development Monitoring and Evaluation Report	http://www.gov.cn/zhengce/zhengceku/2020-05/16/content_5512148.htm
City-level CO_2_ emissions based on the Energy Balance Tables	CEADs	https://www.ceads.net.cn/data/city/
City-level CO_2_ emissions based on the anthropogenic carbon emissions spatial grid dataset	ODIAC	https://db.cger.nies.go.jp/dataset/ODIAC/DL_odiac2020b.html
City-level population, GDP and other relevant data	China City Statistical Yearbook	http://www.stats.gov.cn/tjsj/tjcbw/
New data generated by this study (including provincial-level renewable energy consumption and city-level renewable and fossil energy consumption)	This study	Data S1–S4

**Software and algorithms**		

STATA	17MP	https://www.stata.com/
ArcMap	10.8	https://www.esri.com/en-us/home
MATLAB	R2021a	https://www.mathworks.com/products/matlab.html

### Resource availability

#### Lead contact

Further information and requests for resources should be directed to and will be fulfilled by the lead author, Professor Donglan Zha (zdl@nuaa.edu.cn).

#### Materials availability

This study did not generate new unique materials.

### Method details

#### Data sources

##### Data sources for the calculation of provincial-level renewable energy consumption

The estimation of provincial-level renewable energy consumption requires four types of basic data, namely, renewable energy generation, electricity generation, electricity imports from other provinces, and electricity exports to other provinces. In China, hydroelectricity, wind electricity, and solar electricity are the most important types of renewable energy, while other renewable energy types account for a small share. According to the *Statistical Review of World Energy 2020*, in 2019 hydroelectricity, wind electricity, and solar electricity accounted for 95% of renewable energy power in China, while other renewable energy types accounted for only 5%. Moreover, the *China Electric Power Yearbook* includes data on the production of hydroelectricity, wind electricity, and solar electricity only at the provincial-level. Therefore, the total production of hydroelectricity, wind electricity, and solar electricity was used to represent the renewable energy production. Renewable energy generation data were taken from the *China Electric Power Yearbook*. More in detail, the hydroelectricity generation in Tianjin in 2008 and Shandong in 2005 were calculated according to the average value interpolation method. The development of solar electricity in China was relatively late, and during the period 2005-2010, no provinces had solar electricity generation. Therefore, the solar electricity generation in these years was assigned a value of 0. Electricity generation data were retrieved from the National Bureau of Statistics of China (NBSC), and electricity transmission data were taken from the Energy Balance Table of the *China Energy Statistical Yearbook*.

##### Data sources for the compilation of city-level EBTs

The compilation of city-level EBTs requires two types of basic data: provincial-level EBTs and socio-economic factors. Provincial-level EBTs were taken from the *China Energy Statistical Yearbook*, while socio-economic factors were retrieved from the *China City Statistical Yearbook* and the statistical yearbook of each city, including: (a) Urban/rural/provincial population (in 10,000); (b) The GDP of the following sectors: forestry, animal husbandry and fishery; industry; construction; transport, storage and post; wholesale and retail trades, hotels and catering services; and other services (in 100 million yuan).

In this study, cities were defined as prefecture-level administrative units in China. These cities do not include provincial-level municipalities (e.g., Beijing, Shanghai, Tianjin, and Chongqing), but include provincial capitals (e.g., Nanjing and Xi’an). We presented a time series of renewable energy transition of 279 Chinese cities from 2005 to 2019, covering more than 85% of China’s population (compared to the national population from NBSC), more than 84% of GDP (compared to the national GDP from NBSC), and more than 83% of CO_2_ emissions (compared to the national emissions from ODIAC) in 2019. Hence, the dataset built is representative of the entire China.

##### Data sources for historical contributions

The scope of this study covers 279 cities in China in the period from 2005 to 2019. The decomposition of the driving factors of changes in CO_2_ emissions required five types of city-level data: CO_2_ emissions, fossil energy consumption, renewable energy consumption, GDP, and population. Two reliable sources of data exist on city-level CO_2_ emissions in China: the China Emission Accounts and Datasets (CEADs) and the Open-Data Inventory for Anthropogenic Carbon dioxide (ODIAC). The former includes a gradual accounting based on EBTs, while the latter carries out gradual decomposition based on the anthropogenic carbon emissions spatial grid dataset developed by Oda et al.^32^ In relation to time scales, the CEADs released in August 2022 include the CO_2_ emissions of 287 Chinese cities from 2001 to 2019.^14^ However, due to the lack of CO_2_ emissions data for the majority of cities in this dataset (for example, for Xiamen and Xi’an for 2017, and for Shenzhen for 2004-2007 and 2017), it was not possible to obtain city-level CO_2_ emissions data on a complete time scale.

The CO_2_ emissions of ODIAC were calculated based on the fossil-fuel CO_2_ emissions calculated by the Carbon Dioxide Information Analysis Center (CDIAC). Specifically, the city-level carbon emission data of China from 2005 to 2019 could be obtained by tailoring, synthesizing, and extracting the high-resolution spatial grid data (1 km × 1 km) of the ODIAC dataset. Compared to the CEADs database, the ODIAC database allows to determine the CO_2_ emissions of Chinese cities over a complete time scale (i.e., from 2005 to 2019). Therefore, the city-level CO_2_ emission data of ODIAC were employed in this study. ODIAC emissions estimates include CO_2_ emissions from solid fossil fuels, liquid fossil fuels, gas fossil fuels, and cement production. In China, the majority of electricity is generated through the conversion of fossil fuels, especially coal burning. Therefore, to avoid double calculation, we did not consider CO_2_ emissions generated by electricity production.

To ensure the consistency of the data, we compared the city-level CO_2_ emissions from 2005 to 2017, previously published by CEADs, with the data from the ODIAC dataset. The results showed that, although there is a certain degree of difference in the total amount, the two datasets record the same or similar temporal trend in most cities. As for the other data, the consumption of fossil energy and renewable energy at the city level was derived from this study (see STAR Methods details), while GDP and population data were derived from provincial-level statistical yearbooks, China City Statistical Yearbooks, city-level statistical yearbooks.

#### The estimation of city-level renewable energy transition

##### Calculation of provincial-level renewable energy consumption

The National Development and Reform Commission (NDRC) divides the Chinese power grid into six regional power grids, namely North China, Northeast, East China, Central, Northwest, and South regional power grids (see Figure S2 (A)). The electricity produced by each province needs to be integrated into the corresponding regional power grid and redistributed to each province. Therefore, specific profiles of electricity consumption across different provinces have the same share of total electricity consumption. Based on this feature, we were able to distinguish the proportion of renewable electricity consumption from the total electricity consumption.

Moreover, in China, electricity is not only redistributed within regional grids, but also transmitted between regional grids. Therefore, in and out electricity movements need to be considered when estimating provincial renewable energy consumption. The distribution of energy resources in China is uneven, with clear regional heterogeneity. For example, the underdeveloped Northwest region is rich in wind energy; Shanxi and Inner Mongolia are rich in thermal electricity; Sichuan, Yunnan, and Hubei are rich in hydroelectricity; while the developed East region has poor energy resource endowments, and electricity production cannot meet the demand. This is true, for example, for the electricity production in Jiangsu, Guangdong, and Shanghai.

Over the past two decades, the “West-East Power Transmission Project” was launched as a key strategy to fill the gap between power supply and demand in China. Following Huang and Zou,^33^ we integrated the six regional power grids into four regional combined power grids: the Northwest and North China, the East China and Central China, the South, and the Northeast regional power grids.

Based on the abovementioned criteria, the calculation of provincial-level renewable energy consumption was articulated in the following four steps:

(i) Calculation of the exports of renewable energy to other provinces

Following the principle that the electricity production of each province needs to be integrated into the regional power grid, and considering the transmission of electricity among different provinces, the amount of renewable electricity exported to other provinces was calculated as follows:

(Equation 1) $$RES_{i}=ES_{i}\times \left(REP_{i}/EP_{i}\right)$$

where the subscript *i* represents a specific province; and RES, ES, REP, and EP represent the amount of renewable electricity exported to other provinces, the amount of electricity exported to other provinces, renewable electricity production, and electricity production, respectively.

(ii) Calculation of the renewable energy imports from other provinces

In this study, we redistributed electricity among four regional combined power grids. For any given regional power grid, the proportion of renewable electricity in the total electricity imported from other provinces is the same across all provinces. Therefore, the amount of renewable electricity imported from other provinces was calculated as follows:

(Equation 2) $$REL_{i}=\sum_{i=1}^{n_{j}}RES_{i}\times \left(EL_{i}/\sum_{i=1}^{n_{j}}EL_{i}\right)$$

where $n_{j}$ represents the regional combined power grid composed of regional power grid j; and REL and EL represent the amount of renewable electricity imported from other provinces and the amount of electricity imported from other provinces, respectively.

(iii) Calculation of the net inflow of renewable energy from other provinces

Based on the calculations of Equation 1 and Equation 2, the net inflow of renewable energy from other provinces was determined as follows:

(Equation 3) $$NREL_{i}=REL_{i}-RES_{i}$$

where NREL represents the net inflow of renewable energy from other provinces.

(iv) Calculation of the consumption of renewable energy

For any given province, the consumption of renewable energy depends on both the production of renewable energy and the net inflow of renewable energy from other provinces. Therefore, the provincial-level renewable energy consumption (REC) could be calculated as follows:

(Equation 4) $$REC_{i}=NREL_{i}+REP_{i}$$

Currently, the only source available of provincial-level renewable energy consumption data is the National Energy Administration of China, which publishes provincial-level renewable energy consumption data since 2015. To test the consistency between the calculation results in this study and the results of the ***Renewable Energy Power Development Monitoring and Evaluation Report*** (hereinafter referred to as “the Report”), we conducted a comparative analysis of the results in 2019, as shown in Figure S2(C). The provincial-level renewable energy consumption estimated in this study was very close to the consumption included in the Report. In addition to Hubei, the data of other provinces had a high degree of cross-sectional similarity. This indicates that the provincial-level renewable energy consumption estimated in this study had good accuracy.

In particular, in this study, the renewable energy of Hubei province was found to be significantly higher than that in the Report, and was probably overestimated. Taking 2019 as an example, renewable electricity production was 148.757 billion kWh, and the net electricity exports to other provinces were 63.419 billion kWh. Even if all of the net electricity exports to other provinces were composed of renewable electricity, renewable electricity consumption in Hubei would reach at least 85.338 billion kWh; however, in the Report it was reported as equal to 72.1 billion kWh. Therefore, if the data in the ***China Energy Statistical Yearbook*** are objectively correct, the renewable energy consumption of Hubei in the Report may be underestimated.

We further compared the temporal changes to the total amount. The ***Statistical Review of World Energy*** is an authoritative source of data on renewable energy consumption in major countries and regions around the world. This study summed the estimated provincial-level renewable energy consumption data and compared them to China’s renewable energy consumption data published by BP from 2000 to 2019 (see Figure S2(B)). As we can see, the temporal trends of the two sets of data basically coincided from 2000 to 2019, which further supports the scientific validity of the estimation results in this study.

##### Energy balance table and data matching

Data on energy consumption in China can only be traced at the national, provincial, and sectoral levels,^34^ and data for cities are limited. Therefore, to calculate city-level energy consumption we had to use computational modeling techniques. Existing studies proposed a specific procedure to decompose provincial-level data into city-level data.^15^^,^^29^^,^^35^ They are based on the EBT as the most reliable source of data on different types of energy consumption at the provincial level. The EBT is an aggregate summary of “*total primary energy supply*”, “*input and output of transformation*”, “*loss*”, “*total final consumption*”, “*statistical difference*”, and “*total energy consumption*” in one region.

###### Traditional provincial-level EBT

In the traditional provincial-level EBT, data on the production, trade, transformation, and consumption of 30 types of energy are detailed.^36^^,^^29^ In the provincial-level EBT, part of raw coal and crude oil is used for electricity generation and heat generation in the “*Input and Output of Transformation*” stage, and are processed to obtain secondary energy sources, such as coke, electricity, and heat. Therefore, to avoid double calculation of energy consumption, we only focused on final energy consumption, and temporarily ignored intermediate energy consumption. Moreover, to simplify the estimation process, the energy types in the traditional provincial-level EBT were classified into four categories: total coal (including 11 types of coal-related energy), total petroleum products (including 14 types of oil-related energy), natural gas, and electricity. The traditional provincial-level EBT is presented in Table S3.

###### Extended provincial-level EBT

In the traditional provincial-level EBT, only the total amount of electricity consumption can be obtained. Data on renewable electricity consumption, such as from hydroelectric, wind, and solar energy, are not counted in detail. To address this gap, we extended the traditional provincial-level EBT, and subdivided the final electricity consumption into non-renewable electricity and renewable electricity. Specifically, we matched the estimated renewable electricity consumption presented in the STAR Methods with the final electricity consumption in the traditional provincial-level EBT; then, the final electricity consumption was further subdivided into non-renewable electricity, hydroelectricity, wind electricity, and solar electricity. In this way, the energy types in the extended provincial-level EBT were classified into seven categories: total coal, total petroleum products, natural gas, non-renewable electricity, hydroelectricity, wind electricity, and solar electricity. The extended provincial-level EBT is presented in Table S4.

###### Newly developed city-level EBT

In this study, we systematically searched all city-level EBTs, which were found to be covered by the following two cases:

*Case α: city with an EBT*. Only a few cities in China have an EBT for specific years, such as Guangzhou from 2005 to 2013 and Hohhot from 2009 to 2012.

*Case β: city without an EBT*. In China, the majority of cities, such as Xiamen, Nanjing, and Qingdao, have not compiled an EBT.

Therefore, no city in China has a complete EBT for the period from 2005 to 2019. In these cases, we deduced the city-level EBT from its corresponding provincial-level EBT. Technically, we constructed a socio-economic factor, which can be calculated using different indexes, such as GDP and population, as follows:

(Equation 5) $$P=Index_{city}/Index_{province}\times 100\%$$

Through the socio-economic factor P we scaled down the seven types of energy in the expanded provincial-level EBT to the city level. Following Shan et al.,^37^ we assumed that cities and their corresponding provinces have the same sectoral energy intensity, and that cities and their corresponding provinces have the same per capita residential energy consumption. For “*Agriculture*, *Forestry*, *Animal Husbandry and Fishery*”, “*Industry*”, “*Construction*”, “*Transport*, *Storage and Post*”, “*Wholesale and Retail Trades*, *Hotels and Catering Services*”, and “*Other services*”, we used the corresponding GDP to construct the socio-economic factor *P*. For “*residential consumption*”, we used the corresponding urban/rural population to construct the social economic factor *P*. On this basis, city-level final energy consumption could be calculated as follows:

(Equation 6) $$E_{city}=E_{province}\times P$$

where $E_{city}$ and $E_{province}$ represent city-level and provincial-level final energy consumption, respectively. The total final consumption in the newly-developed city-level EBT is presented in Table S5.

#### The decomposition approach for the historical contributions

Existing studies on the driving factors of CO_2_ emissions are usually based on the Kaya identity, and generally focus on factors such as carbon emissions factor, energy structure, energy intensity, output scale, and population size.^22^^,^^38^ In this study, we distinguished fossil energy and renewable energy, and constructed the following expression to characterize the driving factors of China’s city-level CO_2_ emissions:

(Equation 7) $$C=\frac{C}{E_{fossil}}\times \frac{E_{fossil}}{E_{total}}\times \frac{E_{total}}{Y}\times \frac{Y}{P}\times P=CI\times ES\times EI\times PG\times P\;$$

where CI, ES, EI, PG, and P represent carbon emission factor, energy structure, energy intensity, GDP per capita, and population, respectively. Typically, the combustion efficiency of fossil fuels does not change significantly over a short period of time; therefore, the carbon emission factor was assumed to remain constant. Moreover, since the sum of the proportion of fossil energy (i.e., *ES*) and the proportion of renewable energy (i.e., *RET*) is 1, the change in CO_2_ emissions caused by the change of *ES* is equal to the change of CO_2_ emissions caused by the change of *RET*. Therefore, changes in CO_2_ emissions depend on trends in energy structure (i.e. *RET*), energy intensity, output scale, and population size. Equation 7 was further simplified as follows:

(Equation 8) $$\Delta C=C^{T}-C^{0}\;=\Delta ES_{0}^{T}\times \Delta EI_{0}^{T}\times \Delta PG_{0}^{T}\times \Delta P_{0}^{T}\;=\left[\left(-1\right)\times \Delta RET_{0}^{T}\right]\times \Delta EI_{0}^{T}\times \Delta PG_{0}^{T}\times \Delta P_{0}^{T}$$

Equation 8 shows that the change in CO_2_ emissions can be decomposed into four effects, namely renewable energy transition effect (ΔRET), energy intensity effect (ΔEI), economic scale effect (ΔPG) and population scale effect (ΔP). In this study, we focused on the renewable energy transition effect, which represents the amount of CO_2_ emissions reduced by the increase of the proportion of city-level renewable energy consumption in total energy consumption.

Compared to other methods, the Logarithmic Mean Divisia Index (LMDI) method has the properties of time and factor reversibility and zero-value robustness, and is optimal to quantify the impact of driving factors on the change in CO_2_ emissions.^39^ Therefore, we used the LMDI method to calculate the four effects of Equation 8, as follows:

(Equation 9) $$\Delta X_{i}=\sum_{i}\left(C_{i}^{T}-C_{i}^{0}\right)/\left(\ln\;C_{i}^{T}-\ln\;C_{i}^{0}\right)\times \ln\left(X_{i}^{T}/X_{i}^{0}\right)$$

#### Scenario simulation method

The change rates of ES, EI, PG, and P from period t to period t+1 were set as α, β, σ, and γ, respectively. Following Equation 7, the CO_2_ emissions in period t+1 were calculated as follows:

(Equation 10) $$C_{t+1}={ES}_{t+1}\times {EI}_{t+1}\times {PG}_{t+1}\times P_{t+1}\;=\left(1+\alpha \right){ES}_{t}\times \left(1+\beta \right){EI}_{t}\times \left(1+\sigma \right){PG}_{t}\times \left(1+\gamma \right)P_{t}\;=\left(1+\alpha \right)\left(1+\beta \right)\left(1+\sigma \right)\left(1+\gamma \right)C_{t}\;$$

The change rate of CO_2_ emissions was calculated as follows:

(Equation 11) $$\rho_{CO_{2}}=\left(1+\alpha \right)\times \left(1+\beta \right)\times \left(1+\sigma \right)\times \left(1+\gamma \right)-1$$

Changes in CO_2_ emissions are closely related to the change rates of ES, EI, PG, and P.

#### Simulation scenario design

To predict the CO_2_ emissions reduction of China’s city-level renewable energy transitions from 2020 to 2030, three different development scenarios were set, namely the BAU scenario, the S1 scenario, and the S2 scenario. Moreover, to identify the robustness of the simulation results, we extended the time horizon of the scenario simulation to 2035, and compared the results from 2020 to 2030 with those from 2031 to 2035.

##### The BAU scenario

The BAU scenario is based on the characteristics of past development, and assumes that the technology level and policy intensity remain constant over the period 2020-2030, and that no additional emission reductions measures are imposed. According to existing research findings, changes in economic factors have obvious path-dependent inertia characteristics; moreover, more recent changes have greater impacts on the future, while more distant changes will have smaller impacts.^40^ To fully consider the characteristics of China’s “Five-Year Plan” and cyclical adjustment, the maximum and minimum values of the annual average rate of change of all factors from 2020 to 2030 were selected from the maximum and minimum values in the four periods of 2005-2019, 2005-2010, 2011-2015 and 2016-2019.^41^ Since the more recent period was posited as having a greater impact on the future, the average annual growth rate in the most recent period (i.e., 2016-2019) was selected as the median value of the average annual growth rate. The average annual growth rate of all factors under the BAU scenario from 2020 to 2030 is shown in Table S6.

##### The S1 scenario

The S1 scenario is based on two assumptions: (i) policies have a stronger driving effect on renewable energy transition than other factors^42^; and (ii) The 14^th^ Five-Year Plan clearly emphasized that policy will remain the main means to address carbon emissions and promote the development of renewable energy in the period 2020-2030. Therefore, we set the S1 scenario with a view to exploring the forward trajectory of CO_2_ emissions under policy constraints, as well as decomposing the carbon abatement contribution of city-level renewable energy transition.

###### Energy structure

The 14^th^ Five-Year Plan set the target that by 2025, renewable energy consumption will account for about 18% of primary energy consumption, while non-fossil energy will account for about 20% of total energy consumption. Moreover, the “Energy Production and Consumption Revolution Strategy (2016-2030)” (hereinafter referred to as “the Energy Strategy”) delineated several targets, including that by 2030, the proportion of non-fossil energy consumption will be about 25%. In 2020, the proportion of non-fossil energy consumption in China was 15.9%. In other words, this target requires the proportion of non-fossil energy to increase at an average annual growth rate of about 1%. In this context, we set the average annual growth rate of the energy structure as -1%. Considering the effectiveness and uncertainties of policy implementation, the minimum and maximum annual growth rate of the energy mix were adjusted upward and downward by 0.2 percentage points, and were set as -0.8% and -1.2%, respectively. In China, although there is no specific target for renewable energy development in 2035, under the S1 scenario the share of non-fossil energy consumption is expected to continue to grow rapidly from 2030 to 2035. Therefore, we set the average annual growth rate of energy structure from 2031 to 2035 as equal to -1.2%. Consistent with the 2020-2030 period, the minimum and maximum annual growth rates of energy structure were adjusted upward and downward by 0.2%, to be equal to -1.0% and -1.4%, respectively.

###### Energy intensity

Energy intensity is affected by several factors, such as total energy consumption, GDP growth rate, energy policy, and technological level, and its change is complex. According to the China Energy Outlook 2030, the cumulative decline in energy intensity in 2030 compared to 2015 is expected to reach -46%; this means that the average annual growth rate of energy intensity will be about -2.9%. The 14^th^ Five-Year Plan set a 13.5% reduction in energy intensity as one of the main binding targets for economic and social development. Moreover, the Energy Strategy predicted that by 2030, China’s total energy consumption will be controlled below 6 billion tonnes of standard coal, and the average annual growth rate of energy intensity will be between -2.5% and 3.0% under a GDP growth rate of 5%-6%. In this context, we set -2.7% as the median of average annual growth rate of energy intensity, with a range of 0.5 percentage points upward and downward based on the median to obtain the minimum and maximum annual growth rates. According to the 14th Five-Year Plan and the outline of long-term goals for 2035 (hereinafter referred to as “the Outline 2035”), energy intensity will decline from 2031 to 3035. In addition, the 2050 World and China Energy Outlook states that China’s energy consumption will peak around 2035; therefore, the average annual growth rate of energy intensity in the period 2031-2035 is unlikely to exceed that in the period 2020-2030. Hence, we set the average annual growth rate of energy intensity from 2031 to 2035 to -2.5%, while the minimum and maximum annual growth rates were set to -2.0% and -3.0%, respectively.

###### Population

According to the National Population Plan (2016-2030), China’s fertility rate will gradually increase and stabilize at a moderate level after the implementation of the Two-Child Policy, and the total population will reach 1.45 billion in 2030. In 2020, the total population in China was 1.41 billion; this means that the average annual growth rate of the total population will be 0.27% during 2020-2030. To this respect, the National Health and Family Planning Commission’s forecast that the population will reach 1.45 billion by 2030 is consistent. Accordingly, we set the median of average annual growth rate for the period 2020-2030 at 0.27%. Considering the internal driving forces and external conditions of population development, China’s population change will slowly increase and tend to a relatively stable level. Therefore, based on the median of average annual growth rate, we set an upward and downward variation of 0.1% to indicate the minimum and maximum population growth rates. According to the Outline 2035, the population will continue to grow slowly and steadily from 2031 to 2035. Moreover, policy constraints are unlikely to significantly change the growth rate of population in the short term. Therefore, the average annual growth rate of the population in 2031-2035 is consistent with that in 2020-2030.

###### GDP per capita

According to the data from the National Bureau of Statistics of China, the proportion of China’s total export GDP in 2020 and 2021 was 17.84% and 18.86%, respectively, and more than 80% of the domestic production was digested and recycled domestically.^23^ According to the World Trade Organization (WTO), the proportion of China’s international trade to GDP will decrease significantly in the future under the impact of COVID-19.^43^ This indicates that China’s GDP growth will tend to be stable for a long period of time. Despite the impact of the COVID-19 pandemic, GDP grew at an average annual rate of 2.3% in 2020 and 8.1% in 2021, with a two-year average of 5.1%.^23^ Although the 14^th^ Five-Year Plan did not set a specific economic growth target, the average annual growth rate of 5-6% is considered as a relatively stable growth target.^44^ Therefore, we set the average annual growth rate of GDP from 2020 to 2030 at 5.5%, with an upward and downward variation of 0.5% to indicate the maximum and minimum values, namely 5.0% and 6.0%. Combining these data with the average annual growth rate of population from 2020 to 2030, the median, minimum, and maximum values of GDP per capita from 2020 to 2030 were set as equal to 4.12%, 3.72%, and 4.51%, respectively. According to Liu and Chen,^45^ the potential average annual growth rate of China’s GDP in the period 2020-2035 is about 5.3%. Combined with an average annual growth rate of 5.5% in the period 2020-2030, the average annual growth rate of the GDP in the period 2031-2035 is expected to be about 4.9%. Combining these data with the average annual growth rate of population from 2020 to 2030, we set the average annual growth rate of GDP per capita from 2031 to 2035 at 3.72%. Consistent with the 2020-2030 period, the minimum and maximum annual growth rates of GDP per capita were adjusted upward and downward by 0.4%, to be equal to 3.32% and 4.12%, respectively.

##### The S2 scenario

Technological innovation is the most critical driving force of energy conservation, emission reduction, economic growth, and social development.^46^ It is of great significance to achieve technological breakthrough innovation, especially for emerging renewable energy technological innovation.^47^ In this context, we further strengthened the potential growth rates of renewable energy transition, energy intensity, GDP per capita, and population based on the S1 scenario to describe the S2 scenario. In general, different types of technological innovations may have different impacts on economic factors such as renewable energy transition and GDP per capita. Therefore, the S2 scenario mainly focused on the breakthrough of renewable energy technological innovation, and did not cover all technology breakthrough scenarios.

From 2005 to 2019, the number of renewable energy technological patents in 279 Chinese cities increased by an average of 25% annually. More in detail, wind and solar electricity technological patents had an average annual growth rate of 39% and 20%, respectively. However, the low innovation level of energy storage technology is the real bottleneck of large-scale development of city-level renewable energy in China.^48^ Looking at the international experience, to promote the development of energy storage technology, North American and European countries have formulated a series of incentive and subsidy policies, such as the *Better Energy Storage Technology Act*, the *Promoting Grid Storage Act*, and the *Joint Long-Term Storage Act*. By contrast, China’s energy storage industry has grown at a remarkable rate in recent years, despite its late start. According to Wood Mackenzie, China’s energy storage infrastructure is expected to increase by about 25 times by 2024 compared to 2019, which also means that energy storage technology is expected to usher in major breakthroughs and innovations.

If there will be significant breakthroughs in renewable energy technologies from 2020 to 2030, the share of fossil energy consumption is expected to decline further. In the S2 scenario, we assumed that the growth rate of the energy structure is 0.5% higher than that in the S1 scenario, and that the median annual growth rate of energy structure is -1.5% (see Table S6). On this basis, we set an upward and downward variation of 0.2% to indicate the maximum and minimum values, i.e., -1.7% and -1.3%. With the improvement of renewable energy technology and the transition of renewable energy structure, fossil energy consumption is expected to continue to decline, and energy efficiency is expected to be greatly improved. Therefore, the average annual growth rate of energy intensity in the S2 scenario was increased by 0.3% compared to the S1 scenario, and then the median value of the average annual growth rate of energy intensity in the S2 scenario was determined as equal to -3%. On this basis, the maximum and minimum values of the average annual change rate of energy intensity were obtained by setting a range of 0.5% upwards and downwards, i.e., -3.5% and -2.5%. Moreover, after the breakthrough of renewable energy technological innovation, GDP per capita is expected to further increase with the improvement of productivity. Based on the S1 scenario, we increased the average annual growth rate of per capita GDP in the S2 scenario by 0.5%, and then obtained the median value of average annual growth rate of GDP per capita as equal to 4.61%. On this basis, the maximum and minimum annual growth rates of 4.22% and 5.01% were determined by setting an upward and downward variation of 0.4%. A breakthrough in technological innovation cannot directly affect the promulgation and implementation of population policies. Therefore, the average annual growth rate of population in the S2 scenario is consistent with that in the S1 scenario. Consistent with the period 2020-2030, the average annual growth rate of energy structure, energy intensity, and GDP per capita under the S2 scenario from 2031 to 2035 will be equal to 0.5%, 0.3%, and 0.5%, respectively, compared to the S1 scenario. As for population, the average annual growth rate in the S2 scenario is consistent with that in scenario S1 (see Table S6).

## Data Availability

• This study analyzes existing, publicly available data which are listed in the key resources table. The data generated by our analysis can be found in Data S1–S4. • This study does not report original code, which is available for academic purposes from the lead contact. • Any additional information required to reanalyze the data reported in this paper is available from the lead contact upon request.
