# Supplementary material for: China’s ambitious low-carbon goals require fostering city-level renewable energy transitions
Source: iScience. 2023 Feb 24;26(3):106263. doi: 10.1016/j.isci.2023.106263 (PMC10005902; doi:10.1016/j.isci.2023.106263)
Supplement: Document S1. Figures S1 and S2 and Tables S1–S7 [file mmc1.pdf]

iScience, Volume *26*

Supplemental information

China's ambitious low-carbon goals require fostering city-level renewable energy transitions

Guanglei Yang, Guoxing Zhang, Dongqin Cao, Donglan Zha, and Bin Su

Supplemental information

Supplemental Figures: (Figure S1-S2)

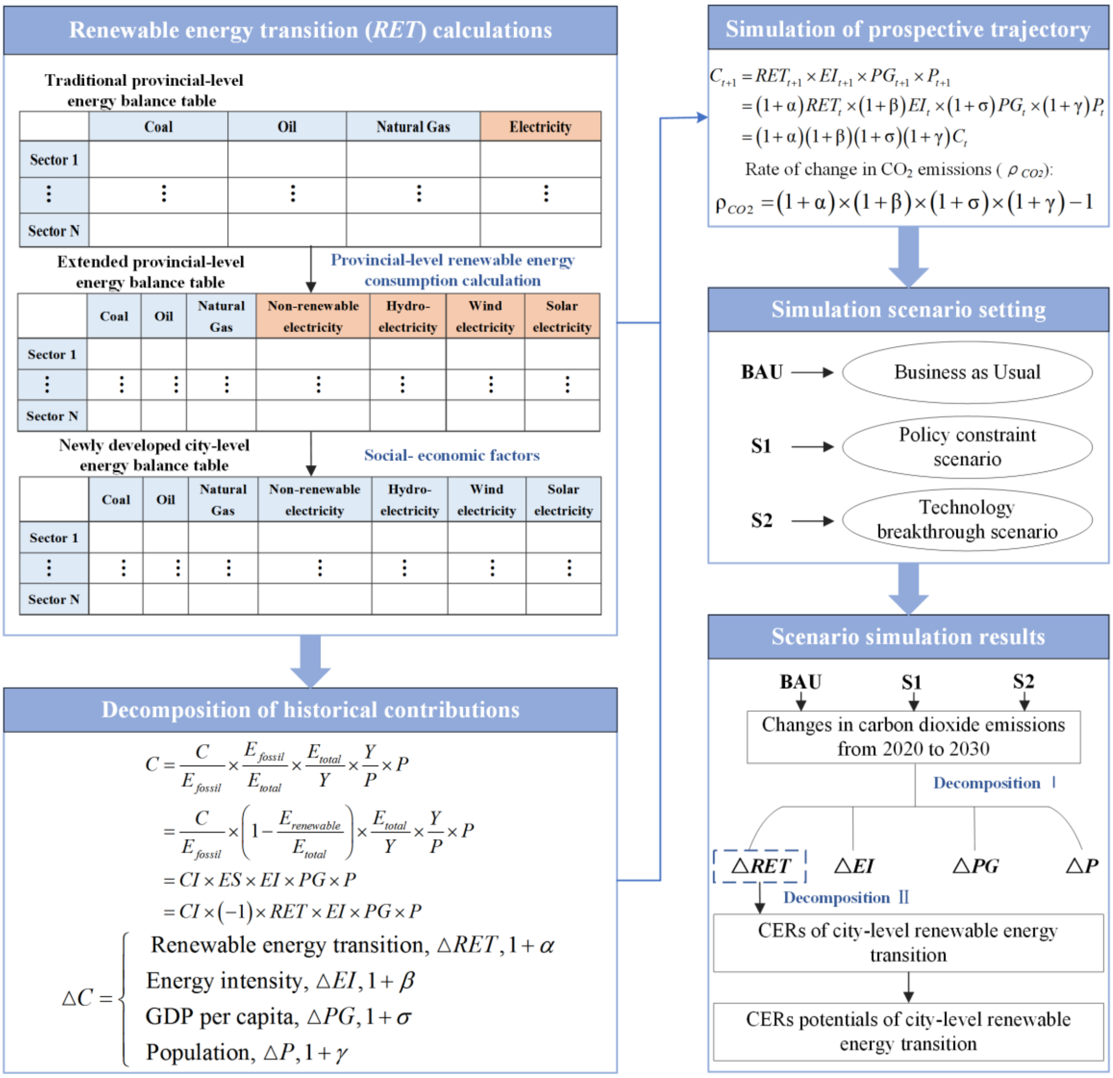

Figure S1. Conceptual framework, related to Figure 1, Figure 2, and Figure 5.

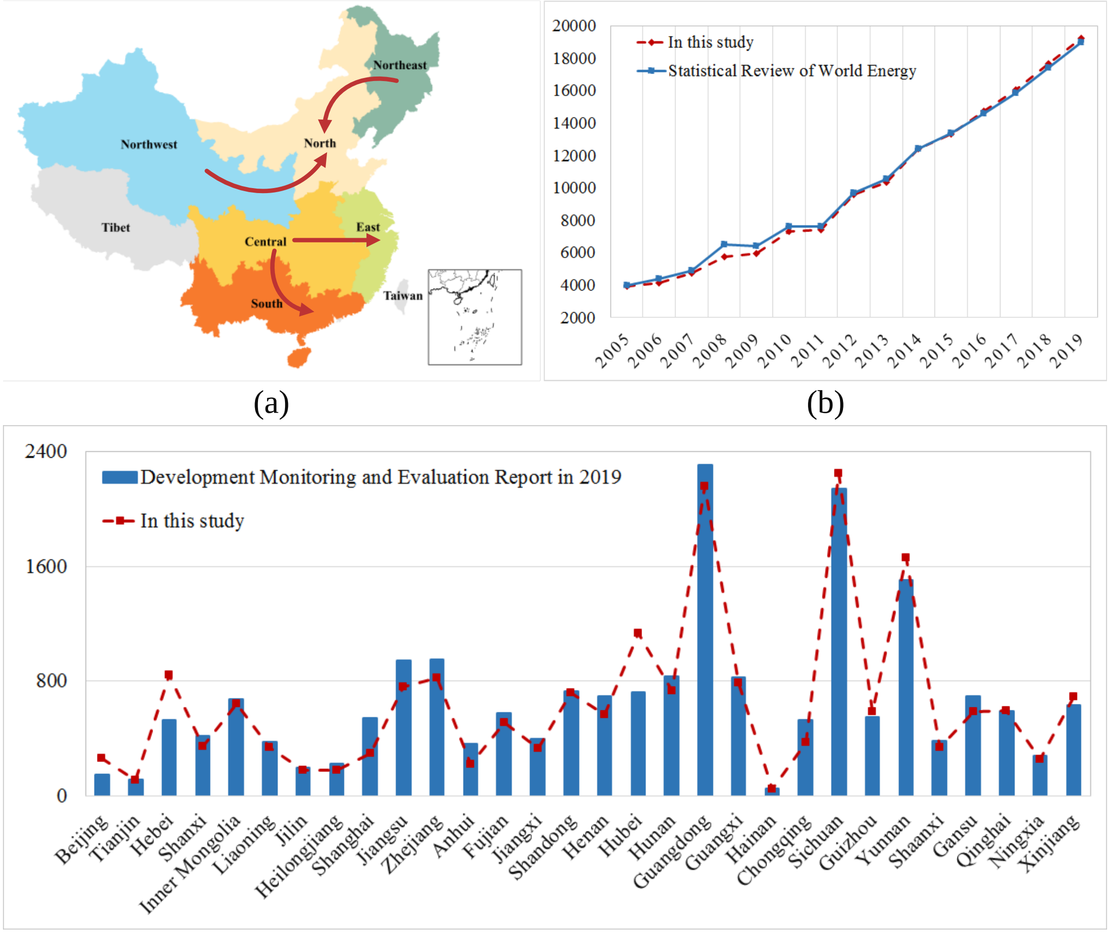

(c)

Figure S2. Regional power grid and power transmission distribution in China (a); Comparison between the results of this study and the Renewable Energy Power Development Monitoring and Evaluation Report, unit: 100 million KWH (b); Comparison between the results of this study and the Renewable Energy Power Development Monitoring and Evaluation Report in 2019, unit: 100 million KWH (c), related to Figure 1.

Supplemental Tables: (Table S1-S7)

Table S1. Representative studies on the impact of renewable energy transition on CO_2 emissions, related to Figure 2 and Figure 4.

Authors	*Renewable energy transition*				*CO_2 emissions*	
	National-level	*Provincial-level*	*City-level*	*Others*	*Historical contribution*	*Prospective trajectory*
Lee et al. (2023)[S1]		√			√	
Murshed (2022)[S2]	√				√	
Yuan et al. (2022)[S3]	√					√
Yang et al. (2022)[S4]	√					√
Fang et al. (2022)[S5]	√				√	
Dong et al. (2022)[S6]	√				√	
Bouyghrissi et al. (2021)[S7]	√				√	
Hamid et al. (2022)[S8]	√				√	
Wang et al. (2022)[S9]	√				√	
De La Peña et al. (2022)[S10]	√					√
Luo et al. (2021)[S11]		√				√
Murshed et al. (2021)[S12]	√				√	
Alqaralleh (2021)[S13]	√				√	
Wei et al. (2021)[S14]	√				√	
Ren et al. (2020)[S15]	√				√	
Wang et al. (2021)[S16]		√			√	
Koengkan & Fuinhas (2020)[S17]	√				√	
Xiao et al. (2019)[S18]		√				√
Usubiaga et al. (2017)[S19]	√				√	
In this study			√		√	√

Table S2. Representative studies of energy and environmental data in Chinese cities, related to Figure 3.

Authors	*Time scale*	*Number of cities*	*Fossil energy consumption*	*Renewable energy consumption*	*CO_2 emissions*
Shan et al. (2022)[S20]	2001-2019	287			√
Liu et al. (2022)[S21]	2003-2019	286	√		
Chen et al. (2022)[S22]	2005-2012	245	√		
Chen et al. (2022)[S23]	1997-2017	336	√		
Cui et al. (2021)[S24]	1999-2014	236	√		
Li et al. (2021)[S25]	2019	336			√
Cai et al. (2019)[S26]	2005	287			√
Wang et al. (2019)[S27]	2013	327			√
Jing et al. (2018)[S28]	2012	41	√		√
Shan et al. (2018)[S29]	2010	182	√		√
Miao (2017)[S30]	2013	216	√		√
Shan et al. (2017)[S31]	2010	24			√
Mi et al. (2016)[S32]	2007	13	√		√
In this study	2005-2019	279	√	√	√

Table S3. The total final consumption in traditional provincial-level energy balance table, related to Figure 1.

Items	Categories			
	Coal Total	Petroleum Products Total	Natural Gas	Electricity
Total Final Consumption	EC_{total_1}	EC_{total_2}	EC_{total_3}	EC_{total_4}
1. Agriculture, Forestry, Animal Husbandry and Fishery	EC_{1-1}	EC_{1-2}	EC_{1-3}	EC_{1-4}
2. Industry	EC_{2-1}	EC_{2-2}	EC_{2-3}	EC_{2-4}
3. Construction	EC_{3-1}	EC_{3-2}	EC_{3-3}	EC_{3-4}
4. Transport, Storage and Post	EC_{4-1}	EC_{4-2}	EC_{4-3}	EC_{4-4}
5. Wholesale and Retail Trades, Hotels and Catering Services	EC_{5-1}	EC_{5-2}	EC_{5-3}	EC_{5-4}
6. Other services	EC_{6-1}	EC_{6-2}	EC_{6-3}	EC_{6-4}
7. Residential	EC_{7-1}	EC_{7-2}	EC_{7-3}	EC_{7-4}

Table S4. The total final consumption in extended provincial-level energy balance table, related to Figure 1.

Items	Categories						
	Coal Total	Petroleum Products Total	Natural Gas	Non-renewable Electricity	Hydroelectricity	Wind Electricity	Solar Electricity
Total Final Consumption	EC_{total_1}	EC_{total_2}	EC_{total_3}	EC_{total_4}	EC_{total_5}	EC_{total_6}	EC_{total_7}
1. Agriculture, Forestry, Animal Husbandry and Fishery	$EC_{1\text{-}1}$	$EC_{1\text{-}2}$	$EC_{1\text{-}3}$	$EC_{1\text{-}4}$	$EC_{1\text{-}2}$	$EC_{1\text{-}3}$	$EC_{1\text{-}4}$
2. Industry	$EC_{2\text{-}1}$	$EC_{2\text{-}2}$	$EC_{2\text{-}3}$	$EC_{2\text{-}4}$	$EC_{2\text{-}5}$	$EC_{2\text{-}6}$	$EC_{2\text{-}7}$
3. Construction	$EC_{3\text{-}1}$	$EC_{3\text{-}2}$	$EC_{3\text{-}3}$	$EC_{3\text{-}4}$	$EC_{3\text{-}5}$	$EC_{3\text{-}6}$	$EC_{3\text{-}7}$
4. Transport, Storage and Post	$EC_{4\text{-}1}$	$EC_{4\text{-}2}$	$EC_{4\text{-}3}$	$EC_{4\text{-}4}$	$EC_{4\text{-}5}$	$EC_{4\text{-}6}$	$EC_{4\text{-}7}$
5. Wholesale and Retail Trades, Hotels and Catering Services	$EC_{5\text{-}1}$	$EC_{5\text{-}2}$	$EC_{5\text{-}3}$	$EC_{5\text{-}4}$	$EC_{5\text{-}5}$	$EC_{5\text{-}6}$	$EC_{5\text{-}7}$
6. Other services	$EC_{6\text{-}1}$	$EC_{6\text{-}2}$	$EC_{6\text{-}3}$	$EC_{6\text{-}4}$	$EC_{6\text{-}5}$	$EC_{6\text{-}6}$	$EC_{6\text{-}7}$
7. Residential	$EC_{7\text{-}1}$	$EC_{7\text{-}2}$	$EC_{7\text{-}3}$	$EC_{7\text{-}4}$	$EC_{7\text{-}5}$	$EC_{7\text{-}6}$	$EC_{7\text{-}7}$

Table S5. The total final consumption in newly developed city-level energy balance table, related to Figure 1.

Items		Categories						
		Coal Total	Petroleum Products Total	Natural Gas	Non-renewable Electricity	Hydroelectricity	Wind Electricity	Solar Electricity
Total Final Consumption		EC_{total_1}	EC_{total_2}	EC_{total_3}	EC_{total_4}	EC_{total_5}	EC_{total_6}	EC_{total_7}
Primary industry	1. Agriculture, Forestry, Animal Husbandry and Fishery	$EC_{1\text{-}1}$	$EC_{1\text{-}2}$	$EC_{1\text{-}3}$	$EC_{1\text{-}4}$	$EC_{1\text{-}2}$	$EC_{1\text{-}3}$	$EC_{1\text{-}4}$
Secondary industry	2. Industry	$EC_{2\text{-}1}$	$EC_{2\text{-}2}$	$EC_{2\text{-}3}$	$EC_{2\text{-}4}$	$EC_{2\text{-}5}$	$EC_{2\text{-}6}$	$EC_{2\text{-}7}$
	3. Construction	$EC_{3\text{-}1}$	$EC_{3\text{-}2}$	$EC_{3\text{-}3}$	$EC_{3\text{-}4}$	$EC_{3\text{-}5}$	$EC_{3\text{-}6}$	$EC_{3\text{-}7}$
Tertiary industry	4. Transport, Storage and Post	$EC_{4\text{-}1}$	$EC_{4\text{-}2}$	$EC_{4\text{-}3}$	$EC_{4\text{-}4}$	$EC_{4\text{-}5}$	$EC_{4\text{-}6}$	$EC_{4\text{-}7}$
	5. Wholesale and Retail Trades, Hotels and Catering Services	$EC_{5\text{-}1}$	$EC_{5\text{-}2}$	$EC_{5\text{-}3}$	$EC_{5\text{-}4}$	$EC_{5\text{-}5}$	$EC_{5\text{-}6}$	$EC_{5\text{-}7}$
	6. Other services	$EC_{6\text{-}1}$	$EC_{6\text{-}2}$	$EC_{6\text{-}3}$	$EC_{6\text{-}4}$	$EC_{6\text{-}5}$	$EC_{6\text{-}6}$	$EC_{6\text{-}7}$
	7. Residential	$EC_{7\text{-}1}$	$EC_{7\text{-}2}$	$EC_{7\text{-}3}$	$EC_{7\text{-}4}$	$EC_{7\text{-}5}$	$EC_{7\text{-}6}$	$EC_{7\text{-}7}$

Table S6. Average annual growth rates of factors under different scenarios from 2020 to 2030 (Unit: %), related to Figure 4.

Year	*Scenarios*	*ES*	*EI*	*PG*	*P*
2020-2030	*BAU*	-0.51 [-0.81, -0.49]	-1.39[-2.11, -1.17]	4.92[3.39, 6.47]	0.55[0.38, 0.81]
	S1	-1.00[-1.20, -0.80]	-2.70[-3.20, -2.20]	4.12[3.72, 4.52]	0.27[0.17, 0.37]
	S2	-1.50[-1.70, -1.30]	-3.00[-3.50, -2.50]	4.62[4.22, 5.02]	0.27[0.17, 0.37]
2031-2035	*BAU*	-0.51 [-0.81, -0.49]	-1.39[-2.11, -1.17]	4.92[3.39, 6.47]	0.55[0.38, 0.81]
	S1	-1.20[-1.40, -1.00]	-2.50[-3.00, -2.00]	3.72[3.32, 4.12]	0.27[0.17, 0.37]
	S2	-1.70[-1.90, -1.50]	-2.80[-3.30, -2.30]	4.22[3.82, 4.62]	0.27[0.17, 0.37]

Note: The maximum and minimum values are shown in parentheses.

Table S7. CO_2 emissions from 2030 to 2035 (unit: 100 million tonnes) and the probability of falling below 2030 CO_2 emissions (unit: %), related to Figure 4.

	2030	2031	2032	2033	2034	2035
S1 scenario	93.00	93.05 (47.8)	93.14 (45.5)	93.34 (44.0)	93.78 (41.3)	93.82 (39.5)
S2 scenario	89.45	89.39 (50.2)	89.15 (52.0)	88.90 (53.8)	88.70 (55.5)	88.56 (57.0)

Note: Probability values are in parentheses.

Supplemental references

[S1] Lee, C., Zhang, J., & Hou, S. (2023). The impact of regional renewable energy development on environmental sustainability in China. Resources Policy, 80, 103245.

[S2] Murshed, M., Mahmood, H., Ahmad, P., Rehman, A., & Alam, M. (2022). Pathways to Argentina's 2050 carbon-neutrality agenda: the roles of renewable energy transition and trade globalization. Environmental Science and Pollution Research, 29, 29949-29966.

[S3] Yuan, R., Ma, Q., Zhang, Q., Yuan, X., Wang, Q., & Luo, C. (2022). Coordinated effects of energy transition on air pollution mitigation and CO_2 emission control in China. Science of the Total Environment, 841, 156482.

[S4] Yang, Y., Wang, H., Löschel, A., & Zhou, P. (2022). Energy transition toward carbon-neutrality in China: Pathways, implications and uncertainties. Frontiers of Engineering Management, 9, 358-372.

[S5] Fang, G., Wang, L., Gao, Z., Chen, J., & Tian, L. (2022). How to advance China's carbon emission peak? A comparative analysis of energy transition in China and the USA. Environmental Science and Pollution Research, 29, 71487-71501.

[S6] Dong, F., Li, Y., Gao, Y., Zhu, J., Qin, C., & Zhang, X. (2022). Energy transition and carbon neutrality: Exploring the non-linear impact of renewable energy development on carbon emission efficiency in developed countries. Resources, Conservation and Recycling, 177, 106002.

[S7] Bouyghrissi, S., Murshed, M., Jindal, A., Berjaoui, A., Mahmood, H., & Khanniba, M. (2021). The importance of facilitating renewable energy transition for abating CO_2 emissions in Morocco. Environmental Science and Pollution Research, 29, 20752-20767.

[S8] Hamid, I., Alam, M., Kanwal, A., Jena, P., Murshed, M., & Alam, R. (2022). Decarbonization pathways: the roles of foreign direct investments, governance, democracy, economic growth, and renewable energy transition. Environmental Science and Pollution Research, 29, 49816 - 49831.

[S9] Wang, Z., Pham, T., Sun, K., Wang, B., Bui, Q., & Hashemizadeh, A. (2022). The moderating role of financial development in the renewable energy consumption-CO_2 emissions linkage: The case study of Next-11 countries. Energy, 254, 124386.

[S10] De La Peña, L., Guo, R., Cao, X., Ni, X., & Zhang, W. (2022). Accelerating the energy transition to achieve carbon neutrality. Resources, Conservation and Recycling, 177, 105957.

[S11] Luo, S., Hu, W., Liu, W., Xu, X., Huang, Q., Chen, Z., & Lund, H. (2021). Transition pathways towards a deep decarbonization energy system-A case study in Sichuan, China. Applied Energy, 302, 117507.

[S12] Murshed, M., Ahmed, R., Kumpamool, C., Bassim, M., & Elheddad, M. (2021). The effects of regional trade integration and renewable energy transition on environmental quality: Evidence from South Asian neighbors. Business Strategy and the Environment, 30, 4154-4170.

[S13] Alqaralleh, H. (2020). On the nexus of CO_2 emissions and renewable and nonrenewable energy consumption in Europe: A new insight from panel smooth transition. Energy &

Environment, 32, 443-457.

[S14] Wei, Y., Zhao, T., Wang, J., & Zhang, X. (2021). Exploring the impact of transition in energy mix on the CO_2 emissions from China's power generation sector based on IDA and SDA. Environmental Science and Pollution Research, 28, 30858-30872.

[S15] Ren, X., Cheng, C., Wang, Z., & Yan, C. (2020). Spillover and dynamic effects of energy transition and economic growth on carbon dioxide emissions for the European Union: A dynamic spatial panel model. Sustainable Development, 29, 228-242.

[S16] Wang, J., Song, C., & Yuan, R. (2021). CO_2 emissions from electricity generation in China during 1997-2040: The roles of energy transition and thermal power generation efficiency. Science of the Total Environment, 773, 145026.

[S17] Koengkan, M., & Fuinhas, J. (2020). Exploring the effect of the renewable energy transition on CO_2 emissions of Latin American & Caribbean countries. International Journal of Sustainable Energy, 39, 515 - 538.

[S18] Xiao, M., Simon, S., & Pregger, T. (2019). Scenario analysis of energy system transition: A case study of two coastal metropolitan regions, eastern China. Energy Strategy Reviews, 26, 100423.

[S19] Usubiaga, A., Acosta-Fernández, J., McDowall, W., & Li, F. (2017). Exploring the macro-scale CO_2 mitigation potential of photovoltaics and wind energy in Europe's energy transition. Energy Policy, 104, 203-213.

[S20] Shan, Y., Guan, Y., Hang, Y., Zheng, H., Li, Y., Guan, D., Li, J., Zhou, Y., Li, L., & Hubacek, K. (2022). City-level emission peak and drivers in China. Science Bulletin, 67, 1910-1920.

[S21] Liu, Q., Cheng, K., & Zhuang, Y. (2022). Estimation of city energy consumption in China based on downscaling energy balance table. Energy, 256, 124658.

[S22] Chen, H., Liu, K., Shi, T., & Wang, L. (2022). Coal consumption and economic growth: A Chinese city-level study. Energy Economics, 109, 105940.

[S23] Chen, J., Liu, J., Qi, J., Gao, M., Cheng, S., Li, K., & Xu, C. (2022). City- and county-level spatio-temporal energy consumption and efficiency datasets for China from 1997 to 2017. Scientific Data, 9, 101. https://doi.org/10.1038/s41597-022-01240-6.

[S24] Cui, W., Li, J., Xu, W.A., & Güneralp, B. (2021). Industrial electricity consumption and economic growth: A spatio-temporal analysis across prefecture-level cities in China from 1999 to 2014. Energy, 222, 119932.

[S25] Li, W., Dong, F., & Ji, Z. (2021). Research on coordination level and influencing factors spatial heterogeneity of China's urban CO_2 emissions. Sustainable Cities and Society, 75, 103323.

[S26] Cai, B., Lu, J., Wang, J., Dong, H., Liu, X., Chen, Y., Chen, Z., Cong, J., Cui, Z., Dai, C., Fang, K., Feng, T., Guo, J., Li, F., Meng, F., Tang, W., Wang, G., Xie, Y., & Zhang, J. (2019). A benchmark city-level carbon dioxide emission inventory for China in 2005. Applied Energy,

233-234, 659-673.

[S27] Wang, S., Shi, C., Fang, C., & Feng, K. (2019). Examining the spatial variations of determinants of energy-related CO_2 emissions in China at the city level using Geographically Weighted Regression Model. Applied Energy, 235, 95-105.

[S28] Jing, Q., Bai, H., Luo, Wen., Cai, B., Xu, H. (2018) A top-bottom method for city-scale energy-related CO_2 emissions estimation: A case study of 41 Chinese cities. Journal of Cleaner Production, 202, 444-455.

[S29] Shan, Y., Guan, D., Hubacek, K., Zheng, B., Davis, S., Jia, L., Liu, J., Liu, Z., Fromer, N., Mi, Z., Meng, J., Deng, X., Li, Y., Lin, J., Schroeder, H., Weisz, H., & Schellnhuber, H. (2018). City-level climate change mitigation in China. Science Advances, 4, 6. DOI: 10.1126/sciadv.aaq0390.

[S30] Miao, L. (2017). Examining the impact factors of urban residential energy consumption and CO_2 emissions in China: Evidence from city-level data. Ecological Indicators, 73, 29-37.

[S31] Shan, Y., Guan, D., Liu, J., Mi, Z., Liu, Z., Liu, J., chroeder, H., Cai, B., Chen, Y., Shao, S., & Zhang, Q. (2017). Methodology and applications of city level CO_2 emission accounts in China. Journal of Cleaner Production, 161, 1215-1225.

[S32] Mi, Z., Zhang, Y., Guan, D., Shan, Y., Liu, Z., Cong, R., Yuan, X., & Wei, Y. (2016). Consumption-based emission accounting for Chinese cities. Applied Energy, 184, 1073-1081.
